# Patient safety and health equity of AI-enabled triage tools in primary care: a systematic review

**DOI:** 10.1136/bmjhci-2026-102427

**Published:** 2026-10-28

**Authors:** Aymn Alamoudi, Yanying Gai, Salwa S Zghebi, Evangelos Kontopantelis, Benjamin C Brown

**Affiliations:** 1 Division of Population Health, Health Services Research and Primary Care, School of Health Sciences, Faculty of Biology, Medicine and Health, The University of Manchester, Manchester, UK; 2 Department of Public Health, Health Informatics Program, School of Nursing and Health Sciences, Jazan University, Jazan, Saudi Arabia; 3 Division of Informatics, Imaging and Data Sciences, School of Health Sciences, Faculty of Biology, Medicine and Health, The University of Manchester, Manchester, UK; 4 Division of Family Medicine, Yong Loo Lin School of Medicine, National University of Singapore, Singapore; 5 Centre for Research in Health Systems Performance, National University of Singapore, Singapore

**Keywords:** Artificial intelligence, Primary Health Care, Health Equity, Decision Support Systems, Clinical, Informatics

## Abstract

**Objectives:**

This review synthesised real-world patient safety evidence for artificial intelligence (AI)-enabled triage tools in primary care and mapped whether safety has been examined across sociodemographic groups.

**Methods:**

PubMed, Embase, Web of Science and Scopus were searched (January 2015–November 2025) with citation searching. Prospective studies evaluating AI triage tools in real-world primary care settings were eligible. Quality was assessed using the Mixed Methods Appraisal Tool; equity dimensions were mapped using PROGRESS-Plus. Random-effects meta-analysis pooled comparable proportion-based outcomes.

**Results:**

Four studies were included; all evaluated rule-based or decision-tree tools as no machine learning or generative AI system met the eligibility criteria. Safe triage rates ranged from 81% to 97.6%, under-triage rates ranged from 2% to 17.2% and over-triage rates ranged from 8% to 29%. An exploratory random-effects meta-analysis of three studies (n=1303) gave a pooled safe triage proportion of 0.908 (95% CI 0.319 to 0.995) with substantial heterogeneity (I²=97.0%); given incompatible outcome definitions across studies, these estimates are hypothesis-generating rather than summary measures. No study analysed differential triage accuracy by any sociodemographic factor despite available demographic data.

**Discussion:**

Safety outcomes were confined to technical performance proxies rather than direct measures of harm, and the absence of equity-stratified analysis may reflect a systemic evidence gap.

**Conclusion:**

To our knowledge, this is the first systematic review to examine both real-world patient safety and equity of AI-enabled triage in primary care; findings may not extend to machine learning or generative AI systems. A Safety-Equity Evidence Mapping (SEEM) matrix and an evidence maturity framing are proposed to characterise this evidence and inform emerging consensus on evidence thresholds.

**PROSPERO registration number:**

CRD420251179046.

**WHAT IS ALREADY KNOWN ON THIS TOPIC**

**WHAT THIS STUDY ADDS**

- To our knowledge, this is the first systematic review to synthesise real-world safety evidence for AI-enabled triage in primary care alongside the extent of equity-stratified evaluation.
- Across four studies, none analysed safety by any equity-relevant characteristic; a safety-equity evidence mapping (SEEM) matrix and an evidence maturity framing are proposed to characterise this gap.

**HOW THIS STUDY MIGHT AFFECT RESEARCH, PRACTICE OR POLICY**

- Equity-stratified safety analysis may warrant consideration in the evaluation and monitoring of AI triage tools, and these findings may inform consensus processes, such as PRIMARY-AI, that seek to establish evidence thresholds for AI deployment in primary care.

## Introduction

 The COVID-19 pandemic accelerated digital pathways into general practice, within which artificial intelligence (AI)-enabled triage tools—symptom checkers and chatbots—have moved from pilot into routine use.^1^ Rwanda’s national babyl service delivered nearly 3.9 million AI-supported triage consultations between 2019 and 2023.^2^ Synthesis evidence suggests triage accuracy varies widely between tools, motivating focused evaluation of safety and equity.^3^ In England, online consultation submissions rose from 2.7 million in October 2023 to 8.3 million in October 2025.^4^ These systems increasingly use AI to route patient contacts before clinical involvement, placing automated decision-making at the point of entry to primary care.^5^ The pace of implementation raises the question of whether these tools have been adequately evaluated for patient safety and equitable performance.

AI-enabled triage tools range from rule-based algorithms and decision trees to machine learning classifiers.^6^ Several systematic reviews have examined digital symptom checkers and triage tools.^3 6 7^ None focused specifically on primary care safety or on differential performance across patient subgroups.^3^ A recurrent limitation is reliance on vignette-based evaluations, which may not capture the complexity and demographic diversity of routine presentations. Zeng *et al* recently proposed the PRIMARY-AI framework for AI evaluation in primary care, identifying substantial gaps in fairness, accessibility and people-centredness.^8^ Patient triage was a key application category with no agreed evidence thresholds.

Patient safety, defined by the WHO as avoiding preventable harm during healthcare,^9^ provides the evaluative lens for this review. Cooper *et al*’s classification of patient safety incidents in primary care encompasses near misses, psychological harm and harm of varying severity.^10^ For AI-enabled triage, safety risks may include misclassification—under-triage, where urgent conditions receive insufficient urgency, and over-triage, where patients are directed to a higher level of care than indicated—as well as automation-related errors and sociotechnical failures.

AI models developed or validated on non-representative datasets may perform differently across demographic groups. Such disparities have been identified in clinical large language models.^11^ The PROGRESS-Plus framework^12^ provides a structured approach to identifying equity-relevant dimensions. Despite recognition of potential AI bias, whether real-world evaluations of AI triage in primary care have assessed differential safety across subgroups remains unclear.

Patient safety and health equity have each been examined in isolation but not, to our knowledge, jointly for AI-enabled triage in primary care. This review therefore aims to: (1) synthesise patient safety evidence for AI-enabled triage tools in primary care; (2) map the extent to which health equity has been assessed using the PROGRESS-Plus framework; and (3) describe levels of evidence maturity across included studies. A companion viewpoint provides the conceptual rationale underpinning this review.^13^

## Methods

This systematic review is reported in accordance with the Preferred Reporting Items for Systematic Reviews and Meta-Analyses (PRISMA) 2020 guidelines (checklist in online supplemental file 2).^14^ The protocol was prospectively registered (PROSPERO: CRD420251179046).

### Eligibility criteria

Studies were eligible if they reported patient safety outcomes of AI-enabled triage systems used by patients in primary care. Eligible interventions were any AI-enabled triage system—rule-based algorithms, decision trees, machine learning classifiers or generative AI—fully automated or clinician-assisted, at the pre-contact or point-of-contact stage. Rule-based algorithms were included as prespecified in the registered protocol; they represent the dominant technology in primary care AI triage,^3 6 7^ and their exclusion would eliminate most available real-world evidence. Any comparator, including none, was accepted. Outcomes included triage appropriateness, diagnostic accuracy, under-triage and over-triage rates, adverse events and timeliness of care.

Eligible designs were empirical real-world primary care studies with actual patient encounters: randomised controlled trials and prospective cohort, cross-sectional, before-and-after and mixed-methods designs. Retrospective, laboratory, simulation and vignette-based studies, case series, case reports, reviews, protocols, editorials and conference abstracts were excluded.

### Information sources and search strategy

PubMed, Embase (via Ovid), Web of Science Core Collection and Scopus were searched from 1 January 2015 to 6 November 2025. Search strategies are in online supplemental file 1.

Backward and forward citation searches were conducted for all included studies and 10 relevant systematic reviews (online supplemental file 1) completed on 20 January 2026.

### Selection process

Records were screened in Covidence (Veritas Health Innovation, Melbourne, Australia). Title and abstract screening and full-text assessment were conducted independently by two reviewers (AA, YG), with conflicts resolved by a third (SSZ). Full-text exclusions, with reasons, are in online supplemental file 6.

### Data extraction

Data were extracted by one reviewer (AA) using a standardised extraction form and independently verified by a second reviewer (YG). Discrepancies were resolved by discussion and adjudication against original publications. Extraction tables are in online supplemental file 5.

### Quality assessment

Methodological quality was assessed using the Mixed Methods Appraisal Tool^15^ as prespecified in the registered protocol.^15^ Assessment was conducted by one reviewer (AA) and verified by a second (YG); tables are in online supplemental file 4.

Three studies evaluated performance against a clinical reference standard, resembling diagnostic test accuracy designs.^16–18^ The Quality Assessment of Diagnostic Accuracy Studies tool (QUADAS-2) was applied as a supplementary sensitivity analysis (online supplemental file 7).^19^ MMAT remained the primary appraisal tool.

### Synthesis methods

Narrative synthesis covered: (1) tool characteristics and study design, (2) patient safety outcomes and (3) health equity evidence. In the Discussion section, outcomes were interpreted through Cooper *et al*’s classification of patient safety incidents in primary care.^10^ The Safety-Equity Evidence Mapping (SEEM) matrix was developed as a descriptive evidence gap map visualising the intersection of safety and equity-dimension reporting across studies; it is not a validated instrument.

Random-effects meta-analysis was prespecified for studies reporting comparable proportion-based triage outcomes against clinician-based reference standards. Studies using non-comparable reference standards were excluded from pooling and reported narratively. Proportions were pooled on the logit scale (inverse variance), with REML between-study variance and Hartung-Knapp-adjusted CIs. Analyses used R V.4.4.2 (meta package 8.2-1).^20^ Heterogeneity was quantified using I² and τ². Generative AI (Claude Opus V.4.8; Anthropic) assisted in preparing the analysis code, reviewed and verified by the authors. Publication bias was not assessed given the limited value of asymmetry tests for proportion outcomes and the small number of pooled studies. Full details, including the rationale for two protocol deviations, are provided in online supplemental file 8.

Health equity was assessed at two levels: whether PROGRESS-Plus-aligned demographic data were collected and reported, and whether differential accuracy or safety was analysed by any equity-relevant factor.

### Patient and public involvement

Patients or the public were not involved in the design, conduct, reporting or dissemination plans of this review.

## Results

### Study selection

Database searches identified 2209 records; after deduplication, 1428 underwent title and abstract screening and 30 proceeded to full-text assessment (figure 1). Citation searching identified 38 additional records, of which 1 met the inclusion criteria.^17^ Four studies were included.

**Figure 1:**
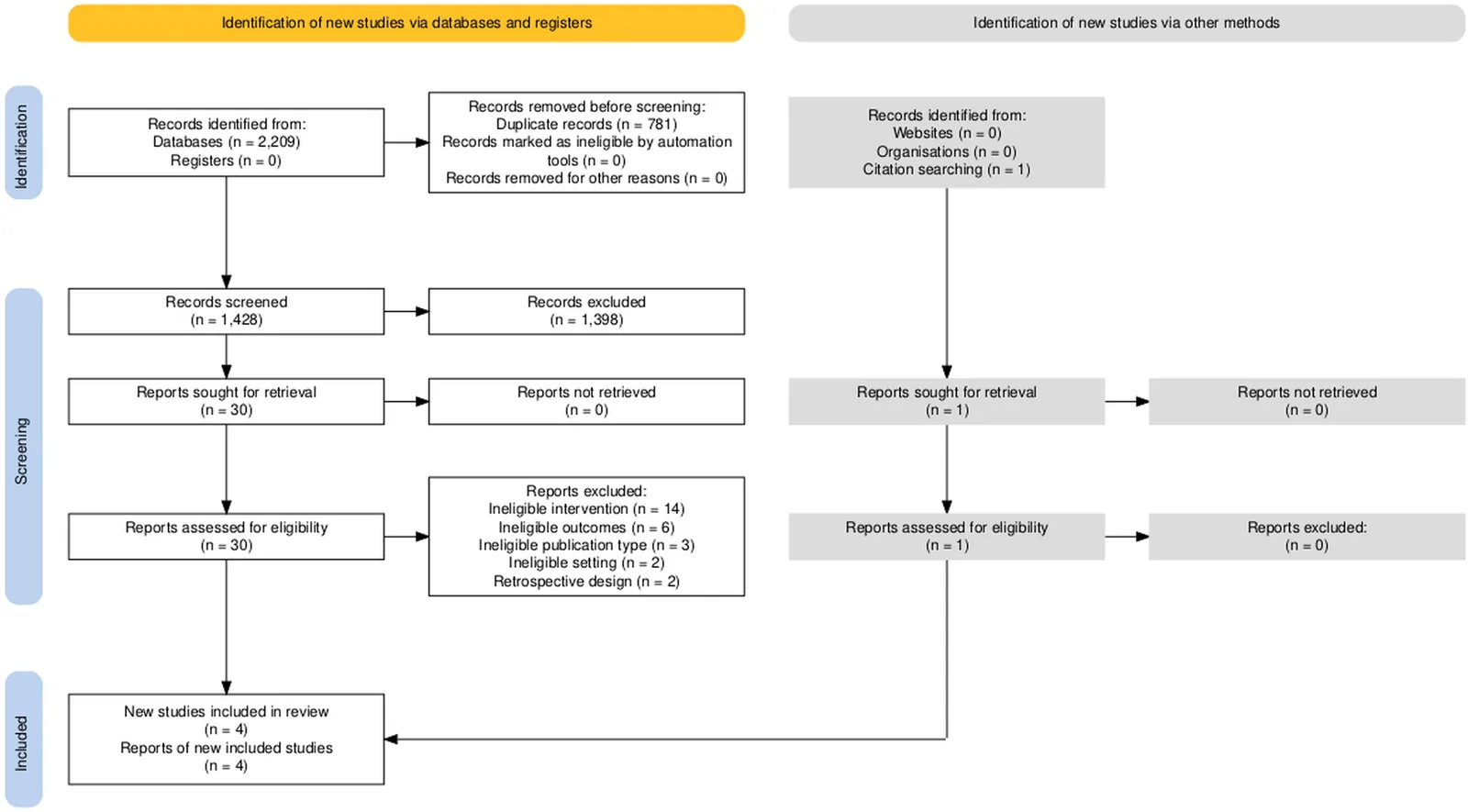
PRISMA (Preferred Reporting Items for Systematic Reviews and Meta-Analyses) 2020 flow diagram. Database searches identified 2209 records across four databases; 30 reports were assessed for eligibility, of which 3 met the inclusion criteria. Citation searching of 10 relevant systematic reviews (listed in online supplemental file 1) identified one additional eligible study, yielding four included studies in total. Generated using the PRISMA 2020 flow diagram tool (https://estech.shinyapps.io/prisma_flowdiagram/).

### Study characteristics

The four studies were conducted in high-income countries and published 2018–2024 (table 1).^16–18 21^ All studies evaluated rule-based or decision-tree triage systems; none employed machine learning or natural language processing. All tools were patient-facing, operating at the pre-contact stage.

**Table 1:** Characteristics of the included studies

Characteristic	Liu *et al*^16^	Chan *et al*^18^	Judson *et al*^21^	Verzantvoort *et al*^17^
Country	Finland	Canada	USA	Netherlands
Design	Prospective clinical validation	Prospective cohort	Prospective observational	Prospective cross-sectional
Setting	18 PHC centres (walk-in)	2 EDs+13 PC clinics (3 provinces)	9 primary care practices (patient portal)	OOH primary care clinic
Total recruited	877	581 (281 ED, 300 PC)	1129 sessions (950 patients)	4456 (of 6194 respondents)
Safety-evaluable	877	300 (PC subgroup)	No single denominator reported	126
Accuracy-evaluable	877	300	No single denominator reported	126
Contributed to meta-analysis	877	300	Not pooled	126
AI tool (type)	Omaolo ESC (rule-based)	Prototype symptom checker (rule-based decision tree)	COVID-19 self-triage tool (rule-based)	‘Should I see a doctor?’ app (decision tree)
Reference standard	Nurse triage assessment	Physician clinical triage	Subsequent care utilisation (48 hours)	Telephone nurse triage
Modality	Web-based	Tablet-based (waiting-room kiosk)	Web-based patient portal (MyChart)	Smartphone app (iOS/Android)
Age	Not collected	Mean 38±16 (ED), 48±18 (PC)	Median 41.6 (IQR 33.6–55.8)	0–12 and >12 years
Sex (% female)	Not collected	63%	62%	66%
Race/ethnicity reported	No	No	Yes (detailed breakdown)	No

ED, emergency department; ESC, electronic symptom checker; OOH, out-of-hours; PC, primary care; PHC, primary healthcare.

Although 7043 participants were recruited across the four studies, far fewer contributed to validated triage outcomes: 877 (Liu *et al*),^16^ the 300 primary-care participants within Chan’s 581^18^ and 126 of Verzantvoort’s 4456 respondents^17^; Judson reported no single accuracy figure.^21^ The exploratory meta-analysis therefore pooled 1303 participants, and the total should not be read as the number contributing to validated primary-care safety outcomes (table 1).

### Quality assessment

Across MMAT domains, all studies had clear research questions and data addressing them. Limitations were concentrated in specific domains rather than captured by an overall score: adjustment for confounders was unclear in two studies,^16 21^ and outcome-data completeness was uncertain in one,^17^ where only 126 of 4456 respondents underwent validated triage comparison. Domain-level judgements are provided in online supplemental file 4.

### Patient safety outcomes

#### Triage appropriateness and diagnostic accuracy

Safe triage rates ranged from 81% (102/126 agreement between app advice and nurse triage)^17^ to 97.6% (856/877; composite definition in table 2).^16^ Judson *et al*^21^ did not report a summary accuracy figure.

**Table 2:** Patient safety outcomes by study

Outcome	Liu *et al*^16^	Chan *et al*^18^	Judson *et al*^21^	Verzantvoort *et al*^17^
Outcome definitions and urgency categories	Safe=exact match, overly conservative, or safe-but-under-conservative relative to nurse triage; unsafe=urgent/on-call (nurse) vs non-urgent/self-care (tool). Categories: P0–P4 and L2–L4.	Safe=exact category match with physician triage; under-triage restricted to missed urgencies with potential harm. Categories: emergency, urgent, routine, home care.	Emergency-level recommendation vs ED attendance within 48 hours. Categories: emergent, non-emergent.	Agreement with telephone nurse triage advice. Categories: call a doctor (general practitioner or out-of-hours) vs don’t call (self-care, wait-and-see); published comparison dichotomised.
Safe triage rate	97.6% (856/877)	73% overall; 85% PC	Not formally reported	81% (102/126)
Exact-match accuracy	53.7%	73% (85% PC)	N/R	81%
Sensitivity	62.6%	100% (emergencies; 10/10)	87.5% (CI 61.7% to 98.5%)	84% (CI 74% to 91%)
Specificity	69.2%	N/R	76.2% (CI 72.9% to 79.5%)	74% (CI 58% to 86%)
Under-triage	17.2% (151/877)	2% (9/581; CI 1 to 3%)	12.5% (2/16 ED)	11% (14/126)
Over-triage	29% (255/877)	27% ED (76/281)	N/R	8% (10/126)
Adverse events	Not assessed	Not assessed	Not assessed	Not assessed

P0–P4=urgency levels requiring professional care (P0=immediate emergency contact). L2–L4=self care levels (Omaolo classification, Liu *et al*).

AI, artificial intelligence; ED, emergency department; N/R, not reported; PC, primary care.

Sensitivity ranged from 62.6% by Liu *et al*^16^ to 100% by Chan *et al* (10/10 emergencies, although underpowered) (table 2).^18^ Judson *et al* reported sensitivity of 87.5% (95% CI 61.7% to 98.5%) and specificity of 76.2% for emergency-level care, against subsequent emergency department (ED) attendance within 48 hours rather than clinician triage.^21^

#### Under-triage

Under-triage rates ranged from 2% (9/581, 95% CI 1% to 3%)^18^ to 17.2% (151/877).^16^ The lower rate reported by Chan *et al*^18^ may reflect their stricter, harm-limited under-triage definition. Liu *et al*^16^ individually reviewed 21 critical assessments and identified no compromised patient safety. Verzantvoort *et al* reported 11% of assessments (14/126) were under-triaged, although none involved life-threatening symptoms.^17^ Judson *et al* did not report under-triage rates; 53% of emergent-triaged patients had no ED visit within 48 hours.^21^

#### Over-triage

Over-triage rates ranged from 8% (10/126)^17^ to 29%.^16^ Chan *et al* reported 27% over-triage to hospital among ED patients (76/281).^18^ Liu *et al*’s over-triage rate was lower than the comparator symptom checker in the same study (Ada: 57.1%).^16^ Verzantvoort *et al* reported 60% of over-triaged users intended to follow the advice.^17^

#### Adverse events and timeliness

No study reported serious adverse events attributable to AI triage. Judson *et al* reported reduced time to appropriate care during the COVID-19 surge.

#### Meta-analysis

Random-effects meta-analysis pooled three studies (n=1303) reporting comparable triage outcomes against clinical reference standards.^16–18^ Judson *et al* was excluded from pooling owing to its different reference standard (care utilisation within 48 hours) and is reported narratively.^21^ These pooled estimates are exploratory: the three studies applied different safe-triage definitions, settings, urgency categories and reference standards, so the results are hypothesis-generating rather than precise summary measures. In a sensitivity analysis re-deriving under-triage and over-triage at a common professional-contact versus self-care threshold across the three studies, heterogeneity decreased (I² 65.8–84.8% vs 89.7–97.1%) and CIs narrowed (under-triage 0.059, 95% CI 0.008 to 0.326; over-triage 0.096, 95% CI 0.047 to 0.186), with conclusions unchanged; results in online supplemental file 8.

The pooled proportions were 0.908 (95% CI 0.319 to 0.995; I²=97.0%) for safe triage, 0.050 (95% CI 0.000 to 0.920; I²=89.7%) for under-triage, and 0.123 (95% CI 0.012 to 0.610; I²=97.1%) for over-triage.

### Health equity analysis

Cells in bold marked “Collected ✗" indicate that demographic data on this PROGRESS-Plus dimension were collected by the study but no differential triage safety analysis was conducted. Dashes (—) indicate the dimension was not collected. Three additional PROGRESS-Plus factors (occupation, religion, social capital) are omitted; none were reported by any study. Full 12-factor matrix in online supplemental file 3.

#### Demographic data reporting

Reporting of PROGRESS-Plus equity dimensions was inconsistent and incomplete across studies (table 3). Age and sex were reported by three studies.^17 18 21^ Liu *et al* collected no patient demographic data.^16^ Race and ethnicity, and socioeconomic status (measured via insurance type), were each reported by one study only.^21^ Education, occupation, religion, social capital and disability status were not reported by any study. Three studies acknowledged digital literacy as a potential barrier; none measured it.

**Table 3:** SEEM matrix

PROGRESS-Plus	Liu *et al* ^16^	Chan *et al*^18^	Judson *et al* ^21^	Verzantvoort *et al*^17^
Place of residence	—	Collected x	—	—
Race/ethnicity	—	—	Collected x	—
Gender/sex	—	Collected x	Collected x	Collected x
Education	—	—	—	—
Socioeconomic status	—	—	Collected x	—
Age	—	Collected x	Collected x	Collected x
Disability	—	—	—	—
Digital literacy	—	—	—	—
Language	—	—	—	—

SEEM, Safety-Equity Evidence Mapping.

#### Differential safety analysis

No included study analysed differential triage accuracy or safety outcomes by any PROGRESS-Plus sociodemographic factor. This absence was observed in the two studies that collected demographic data sufficiently to support such an analysis. Chan *et al* analysed subgroups by care setting but not by patient demographics.^18^ Verzantvoort *et al* analysed age and sex against satisfaction and adherence, but not triage accuracy.^17^ The SEEM matrix (table 3) visualises this absence across studies and PROGRESS-Plus dimensions.

## Discussion

This review examined the patient safety of AI-enabled triage in real-world primary care and whether that safety had been analysed across sociodemographic groups. Four studies met the inclusion criteria. Aggregate safe triage rates ranged from 81% to 97.6%, and no study reported serious adverse events. The pooled safe triage proportion carried wide CIs, reflecting substantial between-study heterogeneity. However, outcomes were confined to technical performance proxies rather than direct measures of patient harm. No included study conducted equity-stratified safety analysis.

The safety-related outcomes reported—agreement, sensitivity, specificity and subsequent care utilisation—are technical proxies of triage performance rather than direct measures of patient safety. Interpreted through Cooper *et al*’s classification, these figures may not capture the full range of safety events.^10^ Under-triage events may be characterised as near misses, and over-triage events as low-severity incidents given psychological burden and unnecessary exposure to acute care. That 60% of over-triaged users intended to follow the advice suggests over-triage may translate into unnecessary healthcare utilisation.^17^ No study followed patients longitudinally or applied a formal harm severity classification; this interpretive classification was applied post hoc. The safety profile of these tools beyond technical performance metrics remains undetermined.

Chambers *et al* assessed digital symptom checkers broadly but did not examine equity dimensions.^7^ Wallace *et al* and Riboli-Sasco *et al* synthesised symptom checker accuracy across settings without demographic stratification or a primary-care safety focus.^3 6^ d’Elia *et al*’s scoping review of AI and health inequities in primary care identified an equity-evidence gap consistent with the present findings.^22^ A recent synthesis of AI diabetic retinopathy screening found that diagnostic performance is setting-dependent and that clinical utility cannot be inferred from sensitivity or specificity alone, cautioning against pooling across pathways with differing reference standards.^23^ This supports the decision not to pool Judson *et al*, whose reference standard differed from those of the other studies.

That no study conducted equity-stratified safety analysis is consistent with emerging evidence. Omar *et al* identified demographic performance disparities in clinical large language models, though direct comparison with rule-based systems warrants caution.^11^ The STANDING Together consensus documented that AI development datasets frequently lack demographic representation and transparency, suggesting the absence of equity-stratified analysis reflects a systemic gap rather than an oversight of these studies.^24^ The gaps identified here may map onto the Tier 1 fairness requirements of the PRIMARY-AI framework, which proposes that AI tools should demonstrate equitable performance across patient subgroups before deployment.^8^ These findings may inform ongoing international consensus processes, such as PRIMARY-AI, that seek to establish evidence thresholds for AI deployment in primary care.

One in five UK general practitioners were reported to have used generative AI in clinical practice in 2024, suggesting that deployment is outpacing the generation of safety and equity evidence.^25^

All included tools were rule-based or decision-tree systems. This reflects the published studies identified from our searches rather than the full range of tools currently used in practice. Consequently our findings may not generalise to triage systems that use machine learning or generative AI; no prospective real-world evaluations of such systems were identified. The patterns observed suggest a trajectory of evidence maturity in AI triage safety-equity evaluation. We organised the studies along a staged evidence maturity framing (figure 2), informed by clinical AI evaluation frameworks (DECIDE-AI^26^ for early-stage clinical evaluation; STANDING Together for equity reporting^24^). The levels were derived inductively from the included studies; the framing is intended as a heuristic rather than a validated classification system, and its applicability beyond these studies remains to be established.

**Figure 2:**
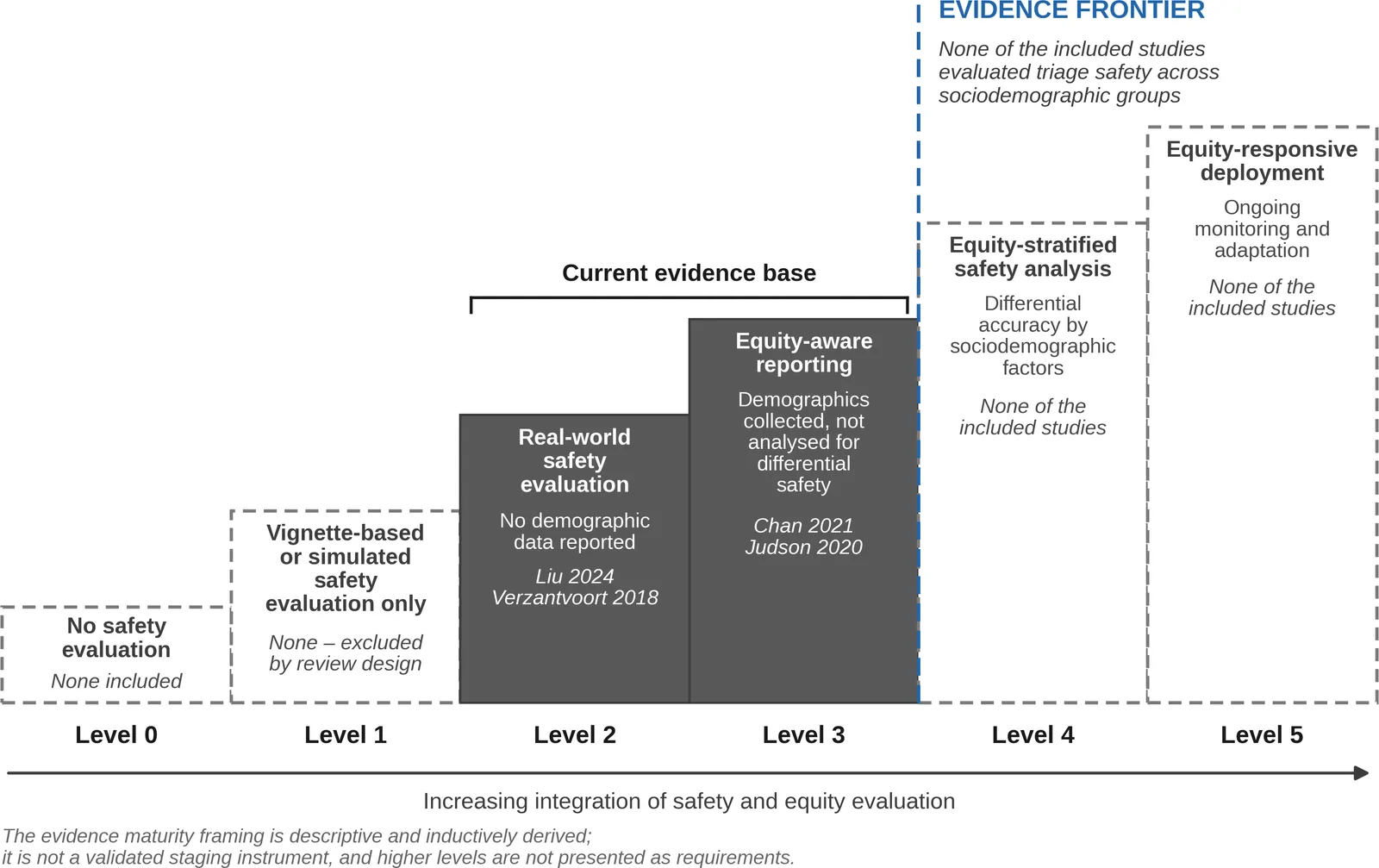
Evidence maturity framing for artificial intelligence triage safety-equity evaluation. Levels 2 and 3 indicate where the included studies currently sit; level 4 denotes the unmet next step, at which differential safety analysis by sociodemographic factors would first be reported. Conceptual grounding and inductive derivation are described in the Discussion section.

The included studies ranged from level 2 (real-world safety evaluation without demographic reporting)^16 17^ to level 3 (equity-aware reporting).^18 21^ The critical frontier is the transition from level 3 to level 4 (equity-stratified safety analysis)—analytical rather than data-related, since level 3 studies already hold the demographic data but do not use it for differential analysis. None of the included studies reached level 5 (equity-responsive deployment).

Most existing evaluations rely on simulated rather than real-world data.^3^ Restricting this review to prospective real-patient studies may strengthen ecological validity while contributing to the small number of studies.

The small number of included studies (n=4) constrains generalisability, although this scarcity is itself a finding reflecting the gap between deployment and prospective real-world safety evidence. Meta-analysis of three studies yielded wide CIs with substantial heterogeneity (I²=89.7–97.1%).^16–18^ The heterogeneity reflects the underlying evidence rather than a property of AI triage as a whole: reviews of AI in more standardised diagnostic settings report minimal heterogeneity,^27^ whereas the varied reference standards and care pathways across the present studies produce substantial between-study variation. Reported performance may also reflect conditions that do not translate to routine deployment: in a diagnostic meta-analysis of biomarker studies, the sampling approach yielding the highest measured association was also the least feasible for routine clinical use.^28^ On QUADAS-2, all three diagnostic-accuracy studies were judged at high overall risk of bias, driven principally by the patient-selection domain, where cohorts showed severity or demographic skew that may limit the representativeness of the reported accuracy; index-test conduct was consistently rated low risk. This review was conducted without patient and public involvement. In an equity-focused review this is itself a limitation: input from patients, particularly those in under-served groups, could have helped prioritise which sociodemographic factors and safety outcomes matter most to those affected, and may have shaped the equity dimensions examined here. The evidence base would benefit from transitioning from technical performance proxies to actual patient outcomes, consistent with the companion Viewpoint’s research agenda.^13^

For policy and practice, deployment of AI triage appears to be outpacing the evidence base, consistent with the PRIMARY-AI outcomes-based standards,^8^ which note that AI is deployed at scale without agreed evaluation frameworks. In England, a national rollout of AI triage through the NHS App was announced in 2026, ahead of published prospective safety or equity evaluation.^29^ Regulators may wish to require equity-stratified safety data in approval and post-market surveillance. Near-miss reporting systems, analogous to existing incident reporting in primary care, may be considered for AI triage tools. Digital triage tools may assume a certain level of health and digital literacy—an assumption that, without active monitoring, may disproportionately affect specific patient populations.^5^ Regulatory approval and demonstrated efficacy do not guarantee sustained equitable access; a regulatorily approved retinal-implant device was later discontinued, leaving previously treated patients without ongoing support.^30^ Prospective safety and equity evaluation of machine learning and generative AI triage tools, as these enter primary care, may be a research priority.

## Conclusions

To our knowledge, this is the first systematic review to examine both real-world patient safety and equity of AI-enabled triage in primary care, although findings may not extend to machine learning or generative AI systems. Such rule-based tools may achieve acceptable aggregate safe triage rates (81–97.6%), based on four heterogeneous studies warranting cautious interpretation. Three gaps persist: safety was measured through technical performance proxies rather than direct patient harm; no study conducted equity-stratified safety analysis, even where demographic data were available; and evidence maturity did not progress beyond equity-aware reporting (level 3). The SEEM matrix and evidence maturity framing were developed post hoc and are descriptive, unvalidated tools; their utility beyond this review remains to be established, and they are not intended to define regulatory, accreditation or deployment thresholds. Until these gaps are addressed, the safety and equitable performance of AI triage in primary care remains undetermined.

## Supplementary material

10.1136/bmjhci-2026-102427 online supplemental file 1

10.1136/bmjhci-2026-102427 online supplemental file 2

10.1136/bmjhci-2026-102427 online supplemental file 3

10.1136/bmjhci-2026-102427 online supplemental file 4

10.1136/bmjhci-2026-102427 online supplemental file 5

10.1136/bmjhci-2026-102427 online supplemental file 6

10.1136/bmjhci-2026-102427 online supplemental file 7

10.1136/bmjhci-2026-102427 online supplemental file 8

## Data Availability

All data relevant to the study are included in the article or uploaded as supplementary information.
