# Supplementary material for: Patient safety and health equity of AI-enabled triage tools in primary care: a systematic review
Source: BMJ Health Care Inform. 2026 Oct 28;33(1):e102427. doi: 10.1136/bmjhci-2026-102427 (PMC13723792; doi:10.1136/bmjhci-2026-102427)
Supplement: Supplementary file 1 [file bmjhci-33-1-s001.pdf]

Online supplemental file 1

Full Search Strategy and Execution Log

Patient Safety and Health Equity of AI-Enabled Triage Tools in Primary Care: A Systematic Review

PROSPERO Registration: CRD420251179046

SM1. Full Search Strategy and Execution Log

This online supplemental file reports the search strategy in two parts: SM1.1 reproduces the search strategy as pre-registered on PROSPERO (CRD420251179046); SM1.2 reports the actual per-database execution performed on 6 November 2025, including the adapted search strings and result counts before and after filters.

SM1.1 Registered Search Strategy

The following reproduces the search strategy as pre-registered on PROSPERO before search execution. The actual per-database execution is reported in Section SM1.2 below.

Title:

Patient Safety and Health Equity of AI-Enabled Triage Tools in Primary Care: A Systematic Review

Databases:

PubMed, Scopus, Web of Science, Embase

Search Period

Studies from 2015 onwards to capture the evolution of AI-enabled triage systems in primary care, aligning with the rapid advancement of machine learning and artificial intelligence technologies in healthcare.

Restricting the search to the past decade ensures a focus on contemporary AI-enabled systems with architectures and performance characteristics relevant to current clinical practice, while excluding outdated systems that may no longer reflect present-day technological capabilities.

Search Terms Development

Search terms were developed through an iterative process informed by preliminary scoping searches and terminology used in previous reviews on AI and digital triage.

The approach focused on identifying combinations of terms related to artificial intelligence, triage, primary care, and patient safety to ensure comprehensive coverage of relevant literature.

For Example :

• AI terminology adapted from Topol EJ (2019). High-performance medicine: the convergence of human and artificial intelligence. Nature Medicine.

• Primary care terminology from Gill PJ, et al. (2014). Identification of children's health care contacts in primary care. Family Practice.

• Online consultation and triage terminology from Darley S, et al. (2022). Understanding how the design and implementation of online consultations affect

primary care quality: systematic review of evidence with recommendations for designers, providers, and researchers. Journal of Medical Internet Research.

• Patient safety terminology from Choudhury A, Asan O (2020). Role of Artificial Intelligence in Patient Safety Outcomes: Systematic Literature Review. JMIR Medical Informatics.

• Health equity terminology from Woolley KE, et al. (2023). Mapping Inequities in Digital Health Technology Within the World Health Organization's European Region Using PROGRESS PLUS: Scoping Review. Journal of Medical Internet Research.

• AI, health equity, and primary care integration from Osonuga A, et al. (2025). Bridging the digital divide: artificial intelligence as a catalyst for health equity in primary care settings. International Journal of Medical Informatics.

Search String (PubMed example):

```
("artificial intelligence" OR "machine learning" OR
"natural language processing" OR "symptom checker" OR
"chatbot" OR "algorithm")
AND
("triage" OR "e-triage" OR "assessment")
AND
("primary care" OR "primary health care" OR
"general practice" OR "family medicine" OR
"family physician*" OR "family practi*")
AND
("safety" OR "accuracy" OR "adverse" OR "appropriate" OR
"error*" OR "harm")
NOT
("simulation" OR "synthetic data" OR "laboratory" OR "vignette")
```

Limits applied:

- Peer-reviewed journal articles only
- Human studies only
- No language or geographical restrictions

Search adaptation:

Equivalent search terms and Boolean logic will be adapted for Scopus, Web of Science, and Embase according to each database’s syntax and field tags.

Additional methods:

- Backward citation searching of included studies
- Forward citation searching of key studies using Google Scholar

Citation search seed reviews (n = 10)

Backward and forward citation searches were run on the four included studies and on the following ten systematic reviews:

1. Darley S, Coulson T, Peek N, et al. Understanding how the design and implementation of online consultations affect primary care quality: systematic review of evidence with recommendations for designers, providers, and researchers. J Med Internet Res 2022;24:e37436. https://doi.org/10.2196/37436

2. Wallace W, Chan C, Chidambaram S, et al. The diagnostic and triage accuracy of digital and online symptom checker tools: a systematic review. npj Digit Med 2022;5:118. https://doi.org/10.1038/s41746-022-00667-w

3. Riboli-Sasco E, El-Osta A, Alaa A, et al. Triage and diagnostic accuracy of online symptom checkers: systematic review. J Med Internet Res 2023;25:e43803. https://doi.org/10.2196/43803

4. Pairon A, Philips H, Verhoeven V. A scoping review on the use and usefulness of online symptom checkers and triage systems: how to proceed? Front Med 2023;9:1040926. https://doi.org/10.3389/fmed.2022.1040926

5. Chambers D, Cantrell AJ, Johnson M, et al. Digital and online symptom checkers and health assessment/triage services for urgent health problems: systematic review. BMJ Open 2019;9:e027743. https://doi.org/10.1136/bmjopen-2018-027743

6. Gottliebsen K, Petersson G. Limited evidence of benefits of patient operated intelligent primary care triage tools: findings of a literature review. BMJ Health Care Inform 2020;27:e100114. https://doi.org/10.1136/bmjhci-2019-100114

7. Saif-Ur-Rahman KM, Islam MS, Alaboson J, et al. Artificial intelligence and digital health in improving primary health care service delivery in LMICs: a systematic review. J Evid Based Med 2023;16:303-20. https://doi.org/10.1111/jebm.12547

8. Farzandipour M, Nabovati E, Sharif R. The effectiveness of tele-triage during the COVID-19 pandemic: a systematic review and narrative synthesis. J Telemed Telecare 2024;30:1367-75. https://doi.org/10.1177/1357633X221150278

9. Abualruz H, Yasin I, Abu Sabra MA, et al. The role of artificial intelligence in enhancing triage decisions in healthcare settings: a systematic review. Applied Nursing Research 2025;86:152024. https://doi.org/10.1016/j.apnr.2025.152024

10. Lammila-Escalera E, Greenfield G, Aldakhil R, et al. Safety and efficacy of digital check-in and triage kiosks in emergency departments: systematic review. J Med Internet Res 2025;27:e69528. https://doi.org/10.2196/69528

Rationale:

The search strategy aims to comprehensively identify studies evaluating AI-enabled triage systems in primary care settings, focusing on patient-safety outcomes and equity-related subgroup differences.

SM1.2 Search Execution Log

The following reports the actual per-database execution of the registered strategy on 6 November 2025. Adapted search strings are presented verbatim for each database, alongside the platform-specific syntax, filters applied, and yields before and after limits.

Table SM1.1 — Search execution summary (6 November 2025)

Database	Initial results	Filters applied	Final results	Export file
**PubMed**	673	Date: 2015–present; ArticleType: Journal; Species: Humans	454	2025-11-06_pubmed_AITriage.nbib (3.3 MB)
**Embase via Ovid**	1,180	Year: 2015–current; Human; Journal	782	2025-11-06_embase_AITriage_01.ris (4.0 MB)
**Web of Science**	368	Timespan: 2015–present; DocType: Article	252	2025-11-06_wos_AITriage.ris (1.0 MB)
**Scopus**	951	PUBYEAR > 2015; DOCTYPE: ar; SRCTYPE: j	721	2025-11-06_scopus_AITriage.ris
**Total**	**3,172**	**—**	**2,209**	**Combined export to Covidence (pre-deduplication)**

Note: Result counts are pre-deduplication. The combined 2,209 records were imported into Covidence, where duplicate removal and screening were performed; the resulting PRISMA 2020 flow is reported in Figure 1 of the main manuscript.

SM1.2.1 PubMed

Platform: PubMed (NCBI). Search date: 6 November 2025. Search interface: PubMed Advanced Search Builder. Result type: peer-reviewed journal articles in humans, 2015 onwards.

```
("artificial intelligence" OR "machine learning" OR "natural language
processing" OR "symptom checker" OR "chatbot" OR "algorithm") AND ("triage" OR
"e-triage" OR "assessment") AND ("primary care" OR "primary health care" OR
"general practice" OR "family medicine" OR "family physician*" OR "family
practi*") AND ("safety" OR "accuracy" OR "adverse" OR "appropriate" OR
"error*" OR "harm") NOT ("simulation" OR "synthetic data" OR "laboratory" OR
"vignette")
```

Filters applied: Date: 2015–present; Article Type: Journal Article; Species: Humans. Initial results: 673. Final results after filters: 454.

SM1.2.2 Embase via Ovid

Platform: Ovid Embase. Search date: 6 November 2025. Search interface: Ovid Advanced Search. The strategy was built line-by-line with intermediate result counts to allow audit and replication.

```
Line 1: ("artificial intelligence" or "machine learning" or "natural language
processing" or "symptom checker" or "chatbot" or "algorithm").mp.
```
→ 924,993 results

```
Line 2: ("triage" or "e-triage" or "assessment").mp.
```
→ 4,373,696 results

```
Line 3: ("primary care" or "primary health care" or "general practice" or "family
medicine" or "family physician*" or "family practi*").mp.
```
→ 416,521 results

```
Line 4: ("safety" or "accuracy" or "adverse" or "appropriate" or "error*" or
"harm").mp.
```
→ 7,057,786 results

```
Line 5: ("simulation" or "synthetic data" or "laboratory" or "vignette").mp.
```
→ 1,959,501 results

```
Line 6: 1 AND 2 AND 3 AND 4
```
→ 1,363 results

```
Line 7: 6 NOT 5
```
→ 1,180 results (initial)

```
Line 8: limit 7 to (human and yr="2015 - current" and journal)
```
→ 782 results (final)

Notes. The .mp. field tag in Ovid Embase searches title, abstract, heading word, drug trade name, original title, device manufacturer, drug manufacturer, device trade name, keyword heading word, floating subheading word, and candidate term word. Truncation (*) was used for "family physician" and "family practi*" to capture variations. Strategy aligns with PROSPERO registration CRD420251179046.*

SM1.2.3 Web of Science

Platform: Web of Science Core Collection. Search date: 6 November 2025. Search interface: Advanced Search; Topic Search (TS=) syntax searches title, abstract, and author keywords.

```
TS=(("artificial intelligence" OR "machine learning" OR "natural language
processing" OR "symptom checker" OR "chatbot" OR "algorithm") AND ("triage" OR
"e-triage" OR "assessment") AND ("primary care" OR "primary health care" OR
"general practice" OR "family medicine" OR "family physician*" OR "family
practi*") AND ("safety" OR "accuracy" OR "adverse" OR "appropriate" OR
"error*" OR "harm") NOT ("simulation" OR "synthetic data" OR "laboratory" OR
"vignette"))
```

Filters applied: Timespan: 2015–present; Document Type: Article. Initial results: 368. Final results after filters: 252.

SM1.2.4 Scopus

Platform: Scopus (Elsevier). Search date: 6 November 2025. Search interface: Advanced Search; TITLE-ABS-KEY syntax searches title, abstract, and keywords.

```
TITLE-ABS-KEY ("artificial intelligence" OR "machine learning" OR "natural
language processing" OR "symptom checker" OR "chatbot" OR "algorithm")
AND TITLE-ABS-KEY ("triage" OR "e-triage" OR "assessment")
AND TITLE-ABS-KEY ("primary care" OR "primary health care" OR "general
practice" OR "family medicine" OR "family physician*" OR "family practi*")
AND TITLE-ABS-KEY ("safety" OR "accuracy" OR "adverse" OR "appropriate" OR
"error*" OR "harm")
AND NOT TITLE-ABS-KEY ("simulation" OR "synthetic data" OR "laboratory" OR
"vignette")
```

Filters applied: PUBYEAR > 2015; Document type: Article (ar); Source type: Journal (j). Initial results: 951. Final results after filters: 721.

Combined yield across the four databases (pre-deduplication): 2,209 records (454 + 782 + 252 + 721). These were exported to Covidence for duplicate removal and screening; the resulting study flow is reported in Figure 1 (PRISMA 2020 flow diagram) of the main manuscript.
