# Supplementary material for: Patient safety and health equity of AI-enabled triage tools in primary care: a systematic review
Source: BMJ Health Care Inform. 2026 Oct 28;33(1):e102427. doi: 10.1136/bmjhci-2026-102427 (PMC13723792; doi:10.1136/bmjhci-2026-102427)
Supplement: Supplementary file 2 [file bmjhci-33-1-s002.pdf]

Online supplemental file 2

PRISMA 2020 Checklist

Patient Safety and Health Equity of AI-Enabled Triage Tools in Primary Care: A Systematic Review
PROSPERO Registration: CRD420251179046

SM2. PRISMA 2020 Checklist

PRISMA 2020 Checklist — Updated April 2026

Systematic Review: Patient Safety and Health Equity of AI-Enabled Triage Tools in Primary Care

PROSPERO: CRD420251179046 | Updated: April 2026

Section	Item #	Checklist Item	Reported?	Location in Manuscript
**TITLE**				
Title	1	Identify the report as a systematic review.	Yes	Title: "Patient Safety and Health Equity of AI-Enabled Triage Tools in Primary Care: A Systematic Review"
**ABSTRACT**				
Abstract	2	See the PRISMA 2020 for Abstracts checklist.	Yes	Abstract: Structured, 250 words, with Objectives, Methods, Results, Discussion and Conclusion sections. Covers objectives, data sources, eligibility criteria, synthesis methods (including exploratory random-effects meta-analysis), results, and conclusions
**INTRODUCTION**				
Rationale	3	Describe the rationale for the review in the context of existing knowledge.	Yes	Introduction, paras 1–4: Digital triage deployment in primary care, evidence gaps in safety and equity, algorithmic bias concerns, PROGRESS-Plus and PRIMARY-AI framing
Objectives	4	Provide an explicit statement of the objective(s) or question(s) the review addresses.	Yes	Introduction, final para: Three objectives stated — (1) synthesise safety evidence, (2) map equity assessment, (3) describe levels of evidence maturity observed across the included studies
**METHODS**				
Eligibility criteria	5	Specify the inclusion and exclusion criteria for the review and how studies were grouped for the syntheses.	Yes	Methods, Eligibility criteria: PICOS criteria. AI-enabled triage systems including rule-based algorithms, ML classifiers, decision-tree tools, neural networks, NLP models, and generative AI systems. Real-world primary care settings. Prospective empirical studies; retrospective and vignette-based excluded
Information sources	6	Specify all databases, registers, websites, organisations, reference lists and other sources searched or consulted to identify studies. Specify the date when each source was last searched or consulted.	Yes	Methods, Information sources and search strategy: PubMed, Embase (Ovid), Web of Science Core Collection, Scopus (searched 1 Jan 2015 to 6 Nov 2025). Backward/forward citation search of included studies + 10 relevant SRs (completed 20 Jan 2026). 38 additional records identified (16 backward + 22 forward)
Search strategy	7	Present the full search strategies for all databases, registers and websites, including any filters and limits used.	Yes	Online supplemental file 1: Full search strategies for all 4 databases
Selection process	8	Specify the methods used to decide whether a study met the inclusion criteria of the review, including how many reviewers screened each record and each report retrieved, whether they worked independently, and if applicable, details of automation tools used in the process.	Yes	Methods, Selection process: Zotero for deduplication; Covidence for screening. Title/abstract screening by AA and YG independently; conflicts resolved by SZ. Full-text screening by AA and YG independently; conflicts resolved through discussion with SZ
Data collection process	9	Specify the methods used to collect data from reports, including how many reviewers collected data from each report, whether they worked independently, any processes for obtaining or confirming data from study investigators, and if applicable, details of automation tools used in	Yes	Methods, Data extraction: Standardised extraction form; AA extracted, YG independently verified. Discrepancies resolved through discussion and adjudication against original papers

Section	Item #	Checklist Item	Reported?	Location in Manuscript
		the process.		
Data items	10a	List and define all outcomes for which data were sought. Specify whether all results that were compatible with each outcome domain in each study were sought.	Yes	Methods, Data extraction: Safety outcomes (triage appropriateness, diagnostic accuracy, under/over-triage, adverse events, timeliness) + equity data (PROGRESS-Plus dimensions)
	10b	List and define all other variables for which data were sought. Describe any assumptions made about any missing or unclear information.	Yes	Methods, Data extraction + Results, Table 1: Study characteristics, AI system type, implementation context, reference standard, sample sizes
Study risk of bias assessment	11	Specify the methods used to assess risk of bias in the included studies, including details of the tool(s) used, how many reviewers assessed each study and whether they worked independently.	Yes	Methods, Quality assessment: MMAT (2018) as primary tool per PROSPERO protocol; AA assessed, YG verified. QUADAS-2 conducted as supplementary sensitivity analysis for 3 studies with diagnostic accuracy designs (online supplemental file 7)
Effect measures	12	Specify for each outcome the effect measure(s) used in the synthesis or presentation of results.	Yes	Methods, Synthesis methods + Results: Individual study proportions (safe triage, under-triage, over-triage rates, sensitivity, specificity) with CIs. Pooled proportions from random-effects meta-analysis (inverse variance pooling on the logit scale, REML, Hartung-Knapp-Sidik-Jonkman adjustment) for 3 studies
Synthesis methods	13a	Describe the processes used to decide which studies were eligible for each synthesis.	Yes	Methods, Synthesis methods: All 4 studies in narrative synthesis. 3 studies (Liu, Chan, Verzantvoort) pooled in random-effects meta-analysis; Judson excluded from pooling due to different reference standard (48h care utilisation vs clinician judgement)
	13b	Describe any methods required to prepare the data for presentation or synthesis.	Yes	Methods, Synthesis methods: Proportions extracted as numerator/denominator for meta-analysis. Outcomes reported as extracted for narrative synthesis
	13c	Describe any methods used to tabulate or visually display results of individual studies and syntheses.	Yes	Methods, Synthesis methods + Results: Tables 1–3 (study characteristics, patient safety outcomes, SEEM Matrix); Figure 2 (Evidence Maturity Framing); online supplemental file 8 (forest plots). Online supplemental file 8 contains full meta-analysis protocol and R code
	13d	Describe any methods used to synthesize results and provide a rationale for the choice(s).	Yes	Methods, Synthesis methods: Narrative synthesis for all 4 studies. Random-effects meta-analysis (inverse variance method on the logit scale, REML, Hartung-Knapp-Sidik-Jonkman adjustment) for 3 comparable studies. Rationale: clinical and methodological heterogeneity warranted random-effects; Judson excluded due to non-comparable reference standard
	13e	Describe any methods used to explore possible causes of heterogeneity among study results.	Yes	Methods, Synthesis methods + Results, Meta-analysis: I^2 and τ^2 reported for each pooled outcome. Heterogeneity explored narratively by comparing settings, tools, populations, reference standards, and outcome definitions
	13f	Describe any sensitivity analyses conducted to assess robustness of the synthesized results.	Yes	Methods, Quality assessment + online supplemental file 7: QUADAS-2 supplementary sensitivity analysis for 3 studies with diagnostic accuracy designs; harmonised-threshold sensitivity analysis re-deriving under- and over-triage at a common professional-contact versus self-care threshold, with leave-one-out influence check (online supplemental file 8)
Reporting bias assessment	14	Describe any methods used to assess risk of bias due to missing results in a synthesis.	Yes	Methods, Synthesis methods + Discussion: Formal assessment (e.g., funnel plot) not feasible due to small number of studies (k=3 in meta-analysis). Reporting bias assessed narratively in Discussion

Section	Item #	Checklist Item	Reported?	Location in Manuscript
Certainty assessment	15	Describe any methods used to assess certainty (or confidence) in the body of evidence for an outcome.	Yes	Methods, Synthesis methods: Formal GRADE not conducted — small number of clinically and methodologically heterogeneous studies precluded meaningful application. Deviation from registered protocol (which specified GRADE-CERQual) acknowledged. Certainty assessed narratively
**RESULTS**				
Study selection	16a	Describe the results of the search and selection process, from the number of records identified in the search to the number of studies included in the review, ideally using a flow diagram.	Yes	Results, Study selection + Figure 1: PRISMA flow diagram. 2,209 records identified → 781 duplicates removed → 1,428 screened → 30 full-text assessed (database) → 27 excluded → 3 included from databases + 1 from citation searching = 4 included
	16b	Cite studies that might appear to meet the inclusion criteria, but which were excluded, and explain why they were excluded.	Yes	Results, Study selection + online supplemental file 6: 27 reports excluded at full-text with reasons (wrong intervention n=14, wrong outcomes n=6, wrong publication type n=3, wrong setting n=2, retrospective design n=2)
Study characteristics	17	Cite each included study and present its characteristics.	Yes	Results, Study characteristics + Table 1: All 4 studies cited with design, country, setting, sample size, AI tool type, reference standard, demographics
Risk of bias in studies	18	Present assessments of risk of bias for each included study.	Yes	Results, Quality assessment: MMAT domain-level judgements for all 4 studies; full domain-level table in online supplemental file 4. online supplemental file 7: QUADAS-2 supplementary analysis for 3 studies
Results of individual studies	19	For all outcomes, present for each study summary statistics and effect estimates with precision.	Yes	Results, Patient safety outcomes + Table 2: Safe triage, sensitivity, specificity, under/over-triage per study with CIs where reported
Results of syntheses	20a	For each synthesis, briefly summarise the characteristics and risk of bias among contributing studies.	Yes	Results, Meta-analysis: Characteristics of 3 pooled studies described (n=1,303 combined); quality context integrated. Judson reported separately with rationale
	20b	Present results of all statistical syntheses conducted.	Yes	Results, Meta-analysis + online supplemental file 8: Pooled safe triage 0.908 (95% CI 0.319–0.995); pooled under-triage 0.050 (95% CI 0.000–0.920); pooled over-triage 0.123 (95% CI 0.012–0.610). I^2 and τ^2 reported per outcome. Online supplemental file 8: full R code and raw data
	20c	Present results of all investigations of possible causes of heterogeneity among study results.	Yes	Results, Meta-analysis: I^2 and τ^2 reported per outcome (safe triage I^2=97.0%; under-triage I^2=89.7%; over-triage I^2=97.1%). Sources of heterogeneity discussed (populations, tools, settings, outcome definitions)
	20d	Present results of all sensitivity analyses conducted to assess the robustness of the synthesized results.	Yes	online supplemental file 7: QUADAS-2 supplementary sensitivity analysis results for 3 studies with diagnostic accuracy designs; harmonised-threshold sensitivity analysis: heterogeneity decreased (I^2 65.8–84.8%) and confidence intervals narrowed (under-triage 0.059, 95% CI 0.008–0.326; over-triage 0.096, 95% CI 0.047–0.186), conclusions unchanged (Results; online supplemental file 8)
Reporting biases	21	Present assessments of risk of bias due to missing results in a synthesis.	Yes	Discussion: No evidence of selective outcome reporting identified. Publication bias cannot be ruled out given small number of included studies. Potential for non-publication of negative AI triage evaluations acknowledged
Certainty of evidence	22	Present assessments of certainty in the body of evidence for each outcome.	Yes	Discussion: Certainty assessed narratively as very low given n=4, substantial heterogeneity (I^2>95%), absence of equity-stratified data, and wide pooled CIs. GRADE not formally applied

Section	Item #	Checklist Item	Reported?	Location in Manuscript
				(deviation acknowledged in Methods)
**DISCUSSION**				
Discussion	23a	Provide a general interpretation of the results in the context of other evidence.	Yes	Discussion: Contextualised against Chambers 2019, Wallace 2022, Omar 2025, PRIMARY-AI (Zeng 2026). Cooper harm classification applied. Evidence maturity framing proposed
	23b	Discuss any limitations of the evidence included in the review.	Yes	Discussion: n=4, all rule-based, proxy safety measures only, no downstream harm data, no equity-stratified analysis, wide CIs in pooled estimates
	23c	Discuss any limitations of the review processes used.	Yes	Discussion: Single reviewer extraction (verified by second); no non-English studies identified (no language restriction imposed); meta-analysis limited to 3 studies with substantial heterogeneity; GRADE not applied
	23d	Discuss implications of the results for practice, policy, and future research.	Yes	Discussion: Research implications (Level 4+ studies, equity-stratified designs, SEEM Matrix). Policy implications (equity-stratified reporting, near-miss systems, STANDING Together, PRIMARY-AI alignment)
**OTHER INFORMATION**				
Registration and protocol	24a	Provide registration information for the review.	Yes	Methods (first para) + Abstract: PROSPERO CRD420251179046
	24b	Indicate where the review protocol can be accessed.	Yes	Methods: Protocol accessible via PROSPERO record. Full search strategy uploaded to PROSPERO and provided in online supplemental file 1
	24c	Describe and explain any amendments to information provided at registration or in the protocol.	Yes	Methods, Synthesis methods: Two protocol deviations acknowledged: (1) GRADE-CERQual not applied (designed for qualitative evidence; inapplicable to quantitative studies included); (2) Random-effects meta-analysis added post-registration (not in original protocol)
Support	25	Describe sources of financial or non-financial support for the review.	Yes	Funding section: No specific funding for this review. Co-author funding disclosed
Competing interests	26	Declare any competing interests of review authors.	Yes	Competing Interests: The authors declare no competing interests
Availability of data	27	Report which of the following are publicly available and where they can be found: template data collection forms; data extracted from included studies; data used for all analyses; analytic code; any other materials used in the review.	Yes	Online supplemental files 1-8: Search strategy (file 1), PRISMA checklist (file 2), PROGRESS-Plus matrix (file 3), MMAT tables (file 4), extraction tables (file 5), excluded studies (file 6), QUADAS-2 analysis (file 7), meta-analysis protocol/data/R code (file 8)

Abbreviations: AI = artificial intelligence; CI = confidence interval; ML = machine learning; NLP = natural language processing; PRIMARY-AI = evaluation framework for artificial intelligence in primary care; PROGRESS-Plus = equity framework (Place of residence, Race/ethnicity/culture/language, Occupation, Gender/sex, Religion, Education, Socioeconomic status, Social capital, plus additional factors); SEEM = Safety-Equity Evidence Mapping.

Reference: Page, M. J. et al. The PRISMA 2020 statement: an updated guideline for reporting systematic reviews. BMJ 372, n71 (2021).
