# Supplementary material for: Patient safety and health equity of AI-enabled triage tools in primary care: a systematic review
Source: BMJ Health Care Inform. 2026 Oct 28;33(1):e102427. doi: 10.1136/bmjhci-2026-102427 (PMC13723792; doi:10.1136/bmjhci-2026-102427)
Supplement: Supplementary file 3 [file bmjhci-33-1-s003.pdf]

Online supplemental file 3

Full PROGRESS-Plus Equity Matrix

Patient Safety and Health Equity of AI-Enabled Triage Tools in Primary Care: A Systematic Review

PROSPERO Registration: CRD420251179046

SM3. Full PROGRESS-Plus Equity Matrix

PROGRESS-Plus Health Equity Data Extraction Matrix

Based on O'Neill et al. (2014) & Cochrane Equity Methods Group Framework

Abbreviations: ED=Emergency Department; PC=Primary Care; Medicaid=US government health insurance for low-income individuals; IQR = interquartile range; LGBTQ+ = lesbian, gay, bisexual, transgender, queer/questioning and other identities.

PROGRESS-Plus Factor	Definition/Examples	Liu 2024 (Finland)	Chan 2021 (Canada)	Judson 2020 (USA)	Verzantvoort 2018 (Netherlands)	Reported n/4
**Place of residence**	Country, region, urban/rural	Yes (18 Primary Health Care centers nationwide)	Yes (3 provinces: Ontario, British Columbia, Yukon)	Yes (Urban 99.6%, Rural 0.4%)	No	3/4
**Race/Ethnicity/Culture/ Language**	Racial/ethnic background, language	No (not collected)	No (not reported)	Yes (48.6% Caucasian, 22.4% Asian, 11.7% Hispanic, 7.5% Non-Hispanic Black, 9.8% Other/Unknown)	No	1/4
**Occupation**	Employment status, job type	No	No	No	No	0/4
**Gender/Sex**	Biological sex, gender identity	No (explicitly stated: not collected)	Yes (63% Female, 37% Male)	Yes (62% Female, 38% Male)	Yes (reported; used in regression for satisfaction)	3/4
**Religion**	Religious affiliation	No	No	No	No	0/4
**Education**	Educational attainment	No	No	No	No	0/4
**Socioeconomic status**	Income, insurance, deprivation indices	No	No	Yes (Insurance: 72.5% Commercial, 13.0% Medicare, 0.0% Medicaid, 0.9% Self-pay)	No	1/4
**Social capital**	Social networks, community support	No	No	No	No	0/4
**PLUS Factors (Additional vulnerable characteristics)**						
**Age**	Age distribution, life stage	No (not collected; only ≥18 years as inclusion criterion)	Yes (Mean: 38±16 yrs ED, 48±18 yrs PC; Range: 16-91)	Yes (Median 41.6 yrs, IQR 33.6-55.8)	Yes (dichotomised 0-12 / >12 years; used in regression)	3/4
**Disability**	Physical, sensory, cognitive disabilities	No	No	No	No	0/4
**Sexual orientation**	Sexual identity, LGBTQ+ status	No	No	No	No	0/4
**Other vulnerabilities**	Digital access barriers, time-dependent factors	Noted as limitation*	No	Noted as limitation**	Smartphone ownership required***	2/4*
**FOOTNOTES:**						
***Liu 2024:**						

1. Users unable to use computer/tablet were excluded from study (noted as limitation)						
2. Full quote (p.19): 'Users who are unable to independently complete the ESC due to technological limitations (such as difficulty using a computer mouse or tablet devices) were excluded from the study'						
**Judson 2020:						
1. Digital divide noted; 0% Medicaid users may indicate limited representation of low-income populations						
2. Full quote (p.865): 'Privately insured patients were overrepresented among the population using the symptom checker compared to the primary care population as a whole, which is consistent with literature on the digital divide.' Tool required patient portal access.						
***Verzantvoort 2018:						
1. App only available on smartphone; users without smartphones excluded by design (digital access barrier)						
2. Sex reported (66% female); age reported (all ages, mostly 19-45 yrs; dichotomised 0-12/>12 in regression)						
EQUITY ANALYSIS						
**Subgroup analysis performed?**		No (by symptoms only)	Yes (ED vs PC setting)	No (descriptive only)	Partial (regression for satisfaction/intention, not triage accuracy)	1/4
**Differential outcomes reported?**		N/A	Yes (PC better accuracy: 85% vs ED 61%)	N/A	No	1/4
**Equity gap identified?**		Digital exclusion noted	Not assessed	Digital divide noted; 0% Medicaid	Smartphone access barrier noted	2/4
Note: Chan 2021's subgroup analysis compared care settings (ED vs PC), not patient demographic characteristics per PROGRESS-Plus framework						
SUMMARY OF FINDINGS						
• Liu 2024: Did not collect age or gender data (explicitly stated)						
• Chan 2021: Collected age/gender but subgroup analysis was by care setting (ED vs PC), not by patient demographics						
• Judson 2020: Reported detailed demographics but analysis was descriptive only						
• Verzantvoort 2018: Collected age/sex; used in regression for satisfaction — but NOT for differential triage accuracy						
• Digital access barriers noted in 2/4 studies but not formally measured or analysed						
• 0% Medicaid users in Judson 2020 may indicate limited representation of disadvantaged groups						
• Factors not reported in any study: Occupation, Religion, Education, Social capital, Disability, Sexual orientation						
• Key finding: No included study analysed differential triage accuracy by any PROGRESS-Plus factor						
• These findings highlight an area for future equity-focused research in AI triage evaluation						

**Framework Reference: O'Neill J, et al. Applying an equity lens to interventions. Int J Equity Health. 2014;13:60.**						

References

1. O'Neill, J. et al. Applying an equity lens to interventions: using PROGRESS ensures consideration of socially stratifying factors to illuminate inequities in health. J. Clin. Epidemiol. 67, 56–64 (2014).
