# Supplementary material for: Patient safety and health equity of AI-enabled triage tools in primary care: a systematic review
Source: BMJ Health Care Inform. 2026 Oct 28;33(1):e102427. doi: 10.1136/bmjhci-2026-102427 (PMC13723792; doi:10.1136/bmjhci-2026-102427)
Supplement: Supplementary file 4 [file bmjhci-33-1-s004.pdf]

Online supplemental file 4

Full MMAT Quality Assessment Tables

Patient Safety and Health Equity of AI-Enabled Triage Tools in Primary Care: A Systematic Review

PROSPERO Registration: CRD420251179046

MMAT Quality Assessment Summary

Study	S1	S2	3.1 Sampling	3.2 Measurement	3.3 Complete data	3.4 Confounders	3.5 Intervention	Score	Rating
**Liu et al. 2024**	Y	Y	Y	Y	Y	CT	Y	4/5	Moderate
**Chan et al.**	Y	Y	Y	Y	Y	Y	Y	5/5	High

**2021**									
**Judson et al. 2020**	Y	Y	Y	Y	Y	CT	Y	4/5	Moderate
**Verzantvoort et al. 2018**	Y	Y	Y	Y	CT	Y	Y	4/5	Moderate

S1 = clear research questions; S2 = data address research questions. Y = Yes; CT = Can’t tell. High = 5/5; Moderate = 4/5.

The criterion rated ‘Can’t tell’ differed across studies. For Liu et al. (2024) and Judson et al. (2020) this was criterion 3.4 (confounders), reflecting limited adjustment for potential confounders including symptom severity variation and absence of a formal comparison group respectively. For Verzantvoort et al. (2018), the ‘Can’t tell’ rating applied to criterion 3.3 (complete outcome data), as only 126 of 4,456 app users had telephone triage validation data available.

SM4. Full MMAT Quality Assessment Tables

The Mixed Methods Appraisal Tool (MMAT) 2018 version was applied as the primary quality-appraisal instrument for the four included studies, in accordance with the registered protocol. This material presents the summary table across all four studies followed by the detailed per-study assessments. Assessments were performed by the first reviewer (AA) and independently verified by the second reviewer (YG), with adjudication by a third reviewer (SSZ) where required.

SM4.1 MMAT Summary Across Included Studies

Study	S1	S2	3.1	3.2	3.3	3.4	3.5	Score	Rating
**Liu et al., 2024**	Y	Y	Y	Y	Y	?	Y	★★★★☆	4/5 (80%)
**Chan et al., 2021**	Y	Y	Y	Y	Y	Y	Y	★★★★★	5/5 (100%)
**Judson et al., 2020**	Y	Y	Y	Y	Y	?	Y	★★★★☆	4/5 (80%)
**Verzantvoort et al., 2018**	Y	Y	Y	Y	?	Y	Y	★★★★☆	4/5 (80%)

Legend & Notes

Colour coding: green = criterion met (Yes); yellow = unclear (Can't tell)

Y = Yes; N = No; ? = Can't tell

Overall Quality Rating:

High = 5 criteria met; Moderate = 4 criteria met; Low = ≤3 criteria met

Screening Questions:

S1 = Are there clear research questions?

S2 = Do the collected data allow to address the research questions?

Methodological Criteria for Non-randomised Quantitative Studies (Category 3):

3.1 = Are the participants representative of the target population?

3.2 = Are measurements appropriate regarding both the outcome and intervention (or exposure)?

3.3 = Are there complete outcome data?

3.4 = Are the confounders accounted for in the design and analysis?

3.5 = During the study period, is the intervention administered (or exposure occurred) as intended?

Study-specific notes:

- Liu et al., 2024: Strong prospective design with large sample (n=877); limitation: no demographic data collected
- Chan et al., 2021: Strong prospective cohort across multiple sites; age/sex collected but no race/socioeconomic status data
- Judson et al., 2020: Strong real-world implementation study with good demographic reporting; limitations: COVID-specific context, short timeframe
- Verzantvoort et al., 2018: Largest sample (n=4,456) but only 126 with diagnostic accuracy validation; strongest quantitative safety metrics (sensitivity 84%, specificity 74%); limitation: small validation subsample

SM4.2 Detailed Per-Study MMAT Assessments

MMAT — Liu 2024

**MMAT Quality Assessment: Liu_2024**		
Study Category: Non-randomised Quantitative		
**SCREENING QUESTIONS**		
**S1. Clear research questions?**	Yes	Clear objective to evaluate Omaolo ESC safety and accuracy
**S2. Data addresses questions?**	Yes	Prospective data directly addresses triage accuracy
**CATEGORY: NON-RANDOMISED QUANTITATIVE**		
**3.1 Participants representative?**	Yes	Walk-in patients at 18 PHC centers across Finland
**3.2 Measurements appropriate?**	Yes	Standard triage categories (MSAH), blinded nurse assessment
**3.3 Complete outcome data?**	Yes	877/877 assessments analysed
**3.4 Confounders accounted for?**	Can't tell	Limited control for symptom severity variation
**3.5 Intervention/exposure as intended?**	Yes	Patients completed ESC before nurse assessment
**OVERALL QUALITY RATING**		
**Rating:**	Moderate (4/5)	
**Reviewer Notes:**		
**Strong prospective design. Limitation: No demographic data collected. Question about whether rule-based system qualifies as 'AI'.**		

MMAT — Chan 2021

MMAT Quality Assessment: Chan_2021		
Study Category: Non-randomised Quantitative		
**SCREENING QUESTIONS**		
**S1. Clear research questions?**	Yes	Clear aim to assess symptom checker safety and accuracy in real patients
**S2. Data addresses questions?**	Yes	Prospective data comparing SC recommendations to physician decisions
**CATEGORY: NON-RANDOMISED QUANTITATIVE**		
**3.1 Participants representative?**	Yes	Walk-in patients at ED and primary care across 3 provinces
**3.2 Measurements appropriate?**	Yes	Physician diagnosis as gold standard, standardised categories
**3.3 Complete outcome data?**	Yes	581/581 included in analysis
**3.4 Confounders accounted for?**	Yes	Separate analysis for ED vs primary care settings
**3.5 Intervention/exposure as intended?**	Yes	Patients blinded to SC recommendations
**OVERALL QUALITY RATING**		
**Rating:**	High (5/5)	
**Reviewer Notes:**		
**Strong prospective cohort design. First study to meet 3 key criteria for SC evaluation. Age/sex collected but no race/SES.**		

MMAT — Judson 2020

MMAT Quality Assessment: Judson_2020		

Study Category: Non-randomised Quantitative		
**SCREENING QUESTIONS**		
**S1. Clear research questions?**	Yes	Clear aim to deploy and evaluate COVID-19 self-triage tool
**S2. Data addresses questions?**	Yes	Real implementation data on tool use and outcomes
**CATEGORY: NON-RANDOMISED QUANTITATIVE**		
**3.1 Participants representative?**	Yes	All UCSF primary care patients with portal access
**3.2 Measurements appropriate?**	Yes	Triage disposition compared to actual care received
**3.3 Complete outcome data?**	Yes	1129 completed sessions analysed
**3.4 Confounders accounted for?**	Can't tell	No formal comparison group design
**3.5 Intervention/exposure as intended?**	Yes	Patients used tool as designed via patient portal
**OVERALL QUALITY RATING**		
**Rating:**	Moderate (4/5)	
**Reviewer Notes:**		
**Strong implementation study. Good demographic data. Limitation: COVID-specific, short timeframe, no formal comparative design.**		

MMAT — Verzantvoort 2018

MMAT Quality Assessment: Verzantvoort_2018		
Study Category: Non-randomised Quantitative		
**SCREENING QUESTIONS**		
**S1. Clear research questions?**	Yes	Clear aim to evaluate whether smartphone self-triage app provides correct advice for acute primary care
**S2. Data addresses questions?**	Yes	Prospective data comparing app advice to telephone nurse triage outcome

**CATEGORY: NON-RANDOMISED QUANTITATIVE**		
**3.1 Participants representative?**	Yes	General public users of self-triage app for acute symptoms in OOH primary care (n=4,456)
**3.2 Measurements appropriate?**	Yes	Telephone nurse triage as reference standard; sensitivity, specificity, PPV, NPV calculated
**3.3 Complete outcome data?**	Can't tell	Only 126 of 4,456 users had telephone triage validation; potential selection bias
**3.4 Confounders accounted for?**	Yes	Multivariate logistic regression for factors associated with intention to follow advice
**3.5 Intervention/exposure as intended?**	Yes	Users completed app independently; nurse unaware of app advice
**OVERALL QUALITY RATING**		
**Rating:**	Moderate (4/5)	
**Reviewer Notes:**		
**Largest sample (n=4,456) but only 126 with diagnostic accuracy validation. Strongest quantitative safety metrics (sens 84%, spec 74%, PPV 88%, NPV 67%, DOR 15). Under-triage 11%. Identified via backward citation search.**		

Abbreviations: AA = Aymn Alamoudi; DOR = diagnostic odds ratio; MMAT = Mixed Methods Appraisal Tool; MSAH = Finnish Ministry of Social Affairs and Health; NPV = negative predictive value; OOH = out-of-hours; PHC = primary health care; PPV = positive predictive value; SC = symptom checker; SES = socioeconomic status; SSZ = Salwa S. Zghebi; UCSF = University of California, San Francisco; YG = Yanying Gai.

References

1. Hong, Q. N. et al. The Mixed Methods Appraisal Tool (MMAT) version 2018 for information professionals and researchers. Educ. Inf. 34, 285–291 (2018).
