# Supplementary material for: Patient safety and health equity of AI-enabled triage tools in primary care: a systematic review
Source: BMJ Health Care Inform. 2026 Oct 28;33(1):e102427. doi: 10.1136/bmjhci-2026-102427 (PMC13723792; doi:10.1136/bmjhci-2026-102427)
Supplement: Supplementary file 5 [file bmjhci-33-1-s005.pdf]

Online supplemental file 5

Full Data Extraction Tables

Patient Safety and Health Equity of AI-Enabled Triage Tools in Primary Care: A Systematic Review

PROSPERO Registration: CRD420251179046

SM5. Full Data Extraction Tables

This material presents the full data extraction for the four included studies. Each field was extracted independently by two reviewers — the first reviewer (AA) and the second reviewer (YG); discrepancies were resolved by discussion, with adjudication by a third reviewer (SSZ) where required. The reconciled final extraction is presented below.

STUDY IDENTIFICATION				
**Field**	**Liu 2024 (#260)**	**Chan 2021 (#690)**	**Judson 2020 (#740)**	**Verzantvoort 2018 (CS-B2)**
**Study ID**	#260	#690	#740	CS-B2
**Full citation**	Liu V, Kaila M, Koskela T. Triage Accuracy and the Safety of User-Initiated Symptom Assessment With an Electronic Symptom Checker in a Real-Life Setting: Instrument Validation Study. JMIR Hum Factors. 2024;11:e55099.	Chan F, Lai S, Pieterman M, Richardson L, Singh A, Peters J, et al. Performance of a new symptom checker in patient triage: Canadian cohort study. PLoS One. 2021;16(12):e0260696.	Judson TJ, Odisho AY, Neinstein AB, et al. Rapid design and implementation of an integrated patient self-triage and self-scheduling tool for COVID-19. J Am Med Inform Assoc. 2020;27(6):860-866.	Verzantvoort NCM, Teunis T, Verheij TJM, van der Velden AW. Self-triage for acute primary care via a smartphone application: Practical, safe and efficient? PLoS One. 2018;13(6):e0199284.
**Country**	Finland	Canada	USA	Netherlands
**Study design**	Clinical validation study in a real-life multicenter primary care setting; prospective	Prospective cohort study	Prospective observational implementation study	Prospective cross-sectional cohort study
**Setting**	18 primary health care (PHC) centres nationwide in Finland; walk-in model primary care. Data collected 1 June 2018 to 31 December 2020.	2 emergency departments (Whitehorse General Hospital, YT; Royal Inland Hospital, Kamloops, BC) and 13 family physician practices across 3 centres in London, Ontario, Canada.	UCSF Health primary care digital front door (patient portal); 9 primary care practices serving ~90,000 patients.	Out-of-hours (OOH) primary care clinic, Utrecht, Netherlands
**Population description**	Real-life patient users attending PHC centres who completed the Omaolo ESC on arrival and then underwent nurse triage. The study population was left unscreened. ~3 in 10 arrived as walk-ins; the rest first contacted by telephone. Adults over 18 only.	Adult patients (≥16 years) self-presenting to emergency departments and family physician clinics with a new medical concern, who completed the prototype symptom checker on a waiting-room tablet before clinical assessment.	Adult empaneled UCSF primary care patients with active MyChart accounts who used the COVID-19 self-triage tool.	General public users of the ‘Should I see a doctor?’ (Moet ik naar de dokter?) smartphone app for acute primary care symptoms.
**Sample size (N)**	877 completed assessments	581 patient encounters	1,129 completed sessions; 950 unique patients	4,456 app users (126 with triage call validation)
**Age group**	Adults only (over 18 recruited); no demographic data collected.	Adults; mean age 38±16 years (ED), 48±18 years (PC); range 16–91	Adults; median 41.6 years (IQR 33.6–55.8)	All ages including children (dichotomised: 0–12 and >12 years); parents used app for children
**AI / TECHNOLOGY**				
**Field**	**Liu 2024 (#260)**	**Chan 2021 (#690)**	**Judson 2020 (#740)**	**Verzantvoort 2018 (CS-B2)**
**Tool / system name**	Omaolo Electronic Symptom Checker (ESC)	Prototype symptom checker (unnamed; designed by author FC)	COVID-19 self-triage and self-scheduling tool (UCSF Coronavirus Symptom Checker)	‘Should I see a doctor?’ (Moet ik naar de dokter?) smartphone app
**Modality**	Web-based electronic symptom checker / self-triage tool	Tablet-based digital symptom checker (waiting-room kiosk use)	Web-based patient portal (MyChart, EHR-tethered)	Smartphone application (iOS/Android; can be used offline)

**AI method (as reported)**	Rule-based algorithm / fixed clinical decision logic based on Duodecim Clinical Decision Support (EBMEDS), using research evidence, probabilities, and expert opinion. Authors explicitly state the system does not employ AI (p.11).	Rule-based decision tree: 247 questions or computational steps manually compiled into a decision tree with recursive elements. NOT machine learning — compiled from medical textbooks and clinical guidelines.	Rule-based algorithm with clinical logic; adapted from CDC guidelines and nursing telephone triage protocols. Over 15 national experts consulted.	Algorithm-based decision tree; fully based on the Dutch Triage System (NTS) and NHG guidelines. CE-marked Class I medical device.
**Workflow position**	Pre-contact triage: patients completed ESC on arrival at PHC centre before nurse assessment.	Pre-contact triage: patients completed checker on waiting-room tablet before clinical encounter.	Pre-contact triage and self-scheduling: patients used tool via patient portal before any clinical contact.	Pre-contact self-triage: patients used app before deciding to contact OOH clinic.
**Human involvement**	Patient-facing automated self-assessment, followed by independent nurse triage as the reference standard. Nurses were initially blinded to ESC output, then allowed to review it and optionally revise their recommendation.	Fully automated patient-facing; physician assessment served as gold standard. Physicians blinded to patient participation.	Primarily automated patient-facing triage with integrated self-scheduling. Clinician involvement occurs only if patients call the hotline/clinic or make an appointment. No proactive outreach.	Fully automated patient-facing; telephone nurse triage served as reference standard.
**Target condition**	General primary care symptoms (multiple symptom categories)	Unrestricted range of symptoms across ED and primary care	COVID-19 symptoms specifically	Acute primary care symptoms (abdominal pain, skin disorders, cough most common)
**Comparator type**	Nurse triage assessment (initially blinded to ESC recommendation).	Physician clinical triage decision (treating physician diagnosis and management plan).	No formal comparator (single-arm implementation study). The study explicitly states inability to compare to a control group.	Telephone nurse triage call outcome.
**SAFETY OUTCOMES**				
**Field**	**Liu 2024 (#260)**	**Chan 2021 (#690)**	**Judson 2020 (#740)**	**Verzantvoort 2018 (CS-B2)**
**Definition of appropriate triage**	Five-level classification: (1) exactly matched; (2) overly conservative but suitable (ESC 1 level higher); (3) safe but under conservative (ESC 1 level lower); (4) overly conservative (ESC 2+ levels higher); (5) potentially unsafe (nurse=urgent, ESC=nonurgent/self-care). Based on Finnish MSAH emergency classification (P0–P4, L2–L4).	Concordance between symptom checker urgency recommendation and physician triage decision. Four categories: emergency, urgency, routine, home-appropriate.	Alignment between self-triage tool recommendation and actual care pathway followed within 48 hours. Four endpoints: emergent, urgent, nonurgent, self-care.	Agreement between app advice (contact GP/OOH clinic, self-care, wait-and-see) and telephone nurse triage outcome (call GP [U1–U4] or wait-and-see [U5]).
**Gold standard / Reference**	Nurse assessment using MSAH national triage criteria.	Physician clinical triage judgement (treating physician diagnosis and management plan).	Subsequent care utilisation within 48 hours (ED visit, in-person, video, telephone, message, or no action). Differs from clinical reference standard used by other studies.	Telephone nurse triage call outcome (using NTS protocol).
**Blinding**	Partial/sequential: nurses did not know ESC result until after completing their own triage assessment. Afterwards, they could view the ESC result and optionally revise.	Patients blinded to symptom checker recommendation; treating physicians blinded to patient participation; categorisation blinded to symptom checker recommendation.	Not reported.	Not explicitly reported; study design implies independent assessment (nurse called user and triaged using NTS protocol without knowledge of app advice).

**Safe triage / Triage appropriateness**	Safe assessments: 97.6% (856/877; 95% CI 95.6–98.0%). Urgency advice agreement reported; moderate-to-good concordance with nurse assessment.	Overall accuracy: 73% (427/581; 95% CI 70–77%). PC accuracy: 85% (255/300; CI 81–89%). ED accuracy: 61% (172/281; CI 55–67%).	Not formally reported as a single safe triage rate. Appropriate triage pathway selection reported descriptively.	81% overall agreement (102/126) between app advice and nurse triage call outcome.
**Sensitivity / Specificity**	Sensitivity 62.6%; Specificity 69.2%. PPV 49.4%; NPV 59.5%; MCC 0.089.	Sensitivity to emergencies: 100% (10/10; CI 70–100%). Sensitivity to urgencies: 90% (73/81; CI 82–95%) hospital; 97% (34/35; CI 85–99%) primary care. PPV reported per setting. Classical specificity not directly reported.	Sensitivity for emergency-level care: 87.5% (95% CI 61.7–98.5%); Specificity: 76.2% (95% CI 72.9–79.5%). Self-care: Sensitivity 53.7% (CI 49.1–58.3%), Specificity 89.5% (CI 84.6–93.2%).	Sensitivity 84% (95% CI 74–91%); Specificity 74% (CI 58–86%); PPV 88% (CI 79–94%); NPV 67% (CI 51–81%); LR+ 3.3; LR− 0.22; DOR 15.
**Under-triage**	17.2% (151/877) undertriaged. 21 critical assessments individually reviewed by expert panel — no compromised patient safety identified.	Under-triage causing potential harm from delay in care: 9 encounters (2%; 95% CI 1–3%). Diagnoses: abscess, phalanx fracture, boxer's fracture, hematoma, hand laceration, metacarpal fracture, toe fracture, foreign body. These were URGENCIES incorrectly sent home.	2 of 16 ED patients (12.5%) were not triaged to emergent by the tool. 92/174 (53%) emergent-triaged patients did not have an ED visit within 48 hours. No specific under-triage rate formally reported.	11% under-triage rate (14/126): app advice 'lower' urgency than nurse triage outcome. Not for life-threatening symptoms. In 6/10 known cases, nurse used a different entry symptom than the patient.
**Over-triage**	29% (255/877) overtriage rate. Evaluations at ratio 100 suitable : 25 overtriage : 22 undertriage. Highest for RTI (44%), heartburn (33%), headache (37%).	Over-triage to hospital: 76/281 ED encounters (27%; CI 22–33%); 20/300 PC encounters (7%; CI 4–10%).	Not formally reported as a rate. Most common triage disposition was self-care (40%). The percentage of emergent dispositions decreased over time as question wording improved.	8% over-triage rate (10/126): app advice 'higher' urgency than nurse triage outcome. 60% of over-triaged users intended to follow the inappropriate advice.
**Adverse events**	No serious adverse events attributed to ESC use. 21 critical cases reviewed, all resolved safely.	No serious adverse events reported.	No safety events reported during implementation period.	No adverse events reported; 65% of users intended to follow app advice.
**Timeliness**	Omaolo ESC has the potential to direct the patient user to the right care pathway	No significant delays to care attributable to tool use	Reduced time to access care; self-scheduling enabled rapid appointment booking	Not directly measured; app designed to facilitate timely OOH consultation decisions
**Safety signal interpretation**	Mostly safe with conservative bias; under-triage rates within acceptable limits. 97.6% safe rate exceeds the 97% threshold set a priori.	Generally safe with conservative over-triage pattern; primary care accuracy (85%) higher than ED (61%). Potential harm in 2–3% of encounters.	Safe in pandemic context; designed for high sensitivity to emergent cases (87.5%) and high specificity for self-care (89.5%). Most self-care patients did not re-contact within 48h.	Safe; sensitivity 84% indicates acceptable detection of patients needing consultation; 11% under-triage warrants attention but not for life-threatening symptoms.
**EQUITY (PROGRESS-Plus)**				

Field	Liu 2024 (#260)	Chan 2021 (#690)	Judson 2020 (#740)	Verzantvoort 2018 (CS-B2)
**Equity domains reported**	None — no demographic data collected (noted as limitation). 'No patient user identifying age or gender data were collected for this study' (p.8).	Age, sex, place of recruitment (3 provinces / 2 EDs + 13 PC clinics).	Age, sex, race/ethnicity (detailed: 48.6% Caucasian, 22.4% Asian, 11.7% Hispanic, 7.5% Non-Hispanic Black, 9.8% Other), marital status, county type (99.6% urban), insurance type (72.5% commercial, 13.0% Medicare, 0.0% Medicaid, 0.9% self-pay).	Age (dichotomised 0–12 / >12 years), sex (66% female).
**Equity analysis conducted**	No — symptoms analysed only, not patient demographics	Partial — separate analysis for ED vs primary care setting (not demographic subgroups)	No — demographics reported descriptively only	Partial — multivariate logistic regression for factors associated with intention to follow advice and satisfaction (age, sex, advice type)

**Equity findings**	N/A — equity dimensions not assessed. Digital literacy noted as potential barrier in Discussion. Introduction cites general literature that healthier/younger/female users use digital services more, but this is NOT a finding of this study.	Primary care setting showed better accuracy (85%) than ED (61%). No demographic-stratified triage accuracy reported.	Detailed demographic data available but not used for differential safety analysis. Portal access may exclude digitally disadvantaged populations. English-only noted as limitation. Privately insured overrepresented (digital divide).	Age and satisfaction associated with intention to follow advice. Triage accuracy was NOT analysed by any demographic subgroup — only overall accuracy reported for n=126.
**IMPLEMENTATION & CONTEXT**				
**Field**	**Liu 2024 (#260)**	**Chan 2021 (#690)**	**Judson 2020 (#740)**	**Verzantvoort 2018 (CS-B2)**
**Implementation & uptake**	Implemented in routine real-life primary care across 18 PHC centres; no formal uptake rate reported	Moderate uptake in routine primary care across 3 provinces	Rapid uptake during COVID-19 surge; 1,129 sessions in implementation period	4,456 study participants (from 6,194 respondents); high user satisfaction (89% neutral to very satisfied)
**Integration into workflow**	Standalone ESC used prior to clinical encounter. The article does not indicate integration into an EHR system.	Standalone tablet-based tool in waiting rooms; not integrated into clinic EHR.	Fully integrated with UCSF MyChart patient portal, scheduling, and EHR. Responses stored in medical record as Patient Self-Triage encounters.	Standalone smartphone app; separate from OOH clinic systems.
**QUALITY & LIMITATIONS**				
**Field**	**Liu 2024 (#260)**	**Chan 2021 (#690)**	**Judson 2020 (#740)**	**Verzantvoort 2018 (CS-B2)**
**Limitations (author-reported)**	No demographic data collected; single-country setting; ESC limited to pre-specified symptoms; selection bias from PHC waiting rooms; adults >18 only (tool designed for >15); users unable to complete independently excluded; serious acute cases rare in setting; mild self-treatable cases may be under-represented; nurse triage may be influenced by user requests (e.g. sick leave); COVID-19 slowed data collection; nurse triage rather than follow-up outcomes used as reference.	Single-country study; clinician judgement as reference standard has own variability; limited to participating sites; very unwell patients less likely to use checker; reproducibility in other countries may be limited by differences in local guidelines, culture, and access; results cannot be generalised to other symptom checkers; unclear whether patients would follow recommendations.	Single health system; pandemic-specific context limits generalisability; no formal comparison group; portal access required; English only; privately insured overrepresented (digital divide); only UCSF medical records reviewed (may miss outside events).	Selection bias (only 126/4,456 users had telephone validation); 9% of validation calls next day (symptoms may have changed); nurse triage not recorded; single OOH clinic.
**Generalisability**	Potentially generalisable to similar real-world primary care digital triage settings, but limited by Finnish single-country context, adult-only recruitment, waiting-room sampling, and exclusion of users unable to complete independently.	Limited by single-country context, challenge of reproducibility across different healthcare systems, and focus on specific clinical sites. Results cannot be generalised to other symptom checkers.	Limited generalisability beyond COVID-19 and US academic health system context.	Applicable to European OOH primary care settings; app covers limited symptom range.
**Risk of bias – measurement**	Appropriate	Appropriate	Appropriate	Appropriate — telephone nurse triage as reference standard
**Risk of bias – completeness**	Complete	Complete	Complete	Partial — only 126/4,456 users had validation calls

**Risk of bias – confounding**	Limited adjustment for confounders (symptom severity variation).	Residual confounding possible; separate ED/PC analysis partially addresses.	No formal comparison group; pre-post design limits causal inference.	Multivariate regression partially addresses for satisfaction and intention to follow advice; no demographic-stratified triage accuracy.
**Overall risk of bias**	Moderate	Low	Moderate	Moderate
**Notes**	Strongest ESC validation in real-world Finnish primary care; key limitation is absence of demographic data	Strongest methodological design (blinded, multi-site, prospective); only study meeting 3 key SC evaluation criteria per authors	Unique implementation study during pandemic; best demographic data of all 4 studies; COVID-specific limits breadth	Largest sample (n=4,456); identified via backward citation search; oldest study (2018); strongest quantitative safety metrics (sens/spec/PPV/NPV)

RECONCILED FINAL VERSION · Reconciled by AA (Reviewer 1) following independent double extraction (AA, YG) · All data verified against original study PDFs

Abbreviations: AA = Aymn Alamoudi; EHR = electronic health record; GP = general practitioner; MCC = Matthews correlation coefficient; NHG = Dutch College of General Practitioners; SSZ = Salwa S. Zghebi; YG = Yanying Gai.
