# Supplementary material for: Patient safety and health equity of AI-enabled triage tools in primary care: a systematic review
Source: BMJ Health Care Inform. 2026 Oct 28;33(1):e102427. doi: 10.1136/bmjhci-2026-102427 (PMC13723792; doi:10.1136/bmjhci-2026-102427)
Supplement: Supplementary file 6 [file bmjhci-33-1-s006.pdf]

Online supplemental file 6

Excluded Studies at Full-Text Review with Reasons

Patient Safety and Health Equity of AI-Enabled Triage Tools in Primary Care: A Systematic Review

PROSPERO Registration: CRD420251179046

Online supplemental file 6

Excluded Studies at Full-Text Screening with Reasons

Patient Safety and Health Equity of AI-Enabled Triage Tools in Primary Care: A Systematic Review

PROSPERO CRD420251179046

Aymn Alamoudi1,2, Yanying Gai1, Salwa S. Zghebi1, Evangelos Kontopantelis3,4, Benjamin C Brown1

[1] Division of Population Health, Health Services Research and Primary Care, School of Health Sciences, Faculty of Biology, Medicine and Health, University of Manchester, Manchester, United Kingdom

[2] Department of Public Health, School of Nursing and Health Sciences, Jazan University, Jazan, Saudi Arabia

[3] Division of Informatics, Imaging and Data Sciences, School of Health Sciences, Faculty of Biology, Medicine and Health, University of Manchester, Manchester, United Kingdom

[4] Division of Family Medicine, Yong Loo Lin School of Medicine, National University of Singapore, Singapore

ORCID: A.A. 0000-0001-8101-8218; Y.G. 0009-0000-2224-4952; S.S.Z. 0000-0002-7978-1094; E.K. 0000-0001-6450-5815; B.C.B. 0000-0001-9975-4782

This online supplemental file lists the 27 full-text articles excluded after eligibility assessment for the systematic review "Patient Safety and Health Equity of AI-Enabled Triage Tools in Primary Care" (PROSPERO: CRD420251179046). Full-text articles were assessed for eligibility independently by both reviewers (AA, YG), with disagreements resolved through discussion with a third reviewer (SSZ). Studies are listed alphabetically by first author with full bibliographic details. Exclusion reasons correspond to the pre-specified eligibility criteria registered on PROSPERO; a summary of the full screening process is presented in the PRISMA 2020 flow diagram (Figure 1).

Summary of exclusion reasons

Reason for exclusion	n
Ineligible intervention	14
Ineligible outcomes	6
Ineligible publication type	3
Ineligible study design	2
Ineligible setting	2
**Total**	**27**

Excluded studies (n = 27)

#	First author(s)	Title	Source	Reason for exclusion
1	Amador-Fernandez, N. et al.	Identification of high-risk patients for referral through machine learning assisting the decision making to manage minor ailments in community pharmacies.	*Front. Pharmacol.* **14**, 1105434 (2023). https://doi.org/10.3389/fphar.2023.1105434	Ineligible setting
2	Brasseur, E., Gilbert, A., Servotte, J. C., Ghuysen, A. & D'Orio, V.	SALOMON, a collaboration model between primary and secondary care for nocturnal emergency calls.	*Rev. Med. Liege* **75**, 83–88 (2020).	Ineligible intervention

#	First author(s)	Title	Source	Reason for exclusion
3	Brasseur, E. et al.	Reliability and validity of an original nurse telephone triage tool for out-of-hours primary care calls: the SALOMON algorithm.	*Acta Clin. Belg.* **77**, 640–646 (2022). https://doi.org/10.1080/17843286.2021.1936353	Ineligible intervention
4	Butler, H. J. et al.	Development of high-throughput ATR-FTIR technology for rapid triage of brain cancer.	*Nat. Commun.* **10**, 4501 (2019). https://doi.org/10.1038/s41467-019-12527-5	Ineligible intervention
5	Churruca, K. et al.	The place of digital triage in a complex healthcare system: an interview study with key stakeholders in Australia's national provider.	*Digit. Health* **9**, 20552076231181201 (2023). https://doi.org/10.1177/20552076231181201	Ineligible intervention
6	Cronin, T. & Travers, J.	Dr AI will see you now: could artificial intelligence replace general practitioners?	*Ir. J. Med. Sci.* **194**, 1353–1358 (2025). https://doi.org/10.1007/s11845-025-04006-2	Ineligible publication type
7	Ellertsson, S., Hlynsson, H. D., Loftsson, H. & Sigurðsson, E. L.	Triaging patients with artificial intelligence for respiratory symptoms in primary care to improve patient outcomes: a retrospective diagnostic accuracy study.	*Ann. Fam. Med.* **21**, 240–248 (2023). https://doi.org/10.1370/afm.2970	Ineligible study design
8	Entezarjou, A., Edstedt-Bonamy, A.-K. E., Benjaminsson, S., Herman, P. & Midlöv, P.	Human- versus machine learning-based triage using digitalized patient histories in primary care: comparative study.	*JMIR Med. Inform.* **8**, e18930 (2020). https://doi.org/10.2196/18930	Ineligible outcomes
9	Evans, H. P. et al.	Automated classification of primary care patient safety incident report content and severity using supervised machine learning (ML) approaches.	*Health Informatics J.* **26**, 3123–3139 (2020). https://doi.org/10.1177/1460458219833102	Ineligible intervention
10	Gams, M. et al.	Evaluating a nationally localized AI chatbot for personalized primary care guidance: insights from the HomeDOCtor deployment in Slovenia.	*Healthcare* **13**, 1843 (2025). https://doi.org/10.3390/healthcare13151843	Ineligible outcomes
11	Giavina-Bianchi, M., Cordioli, E. & Santos, A. P. D.	Accuracy of deep neural network in triaging common skin diseases of primary care attention.	*Front. Med.* **8**, 670300 (2021). https://doi.org/10.3389/fmed.2021.670300	Ineligible intervention
12	Jindal, A. et al.	Usability of an artificially intelligence-powered triage platform for adult ophthalmic emergencies: a mixed methods study.	*Sci. Rep.* **13**, 22490 (2023). https://doi.org/10.1038/s41598-023-49213-y	Ineligible outcomes

#	First author(s)	Title	Source	Reason for exclusion
13	Jorro Barón, F. et al.	Timely identification of deteriorating patients from acute respiratory infections at the primary care level in the COVID-19 era: quality improvement collaborative.	*BMJ Open Qual.* **14**, e003390 (2025). https://doi.org/10.1136/bmjoq-2025-003390	Ineligible intervention
14	Liu, L., Alate, R. & Harrison, J.	Development and validation of PolyScan, an information technology triage tool for older adults with polypharmacy: a healthcare informatics study.	*J. Prim. Health Care* **15**, 215–223 (2023). https://doi.org/10.1071/HC23034	Ineligible intervention
15	Liu, S. et al.	Detecting emergencies in patient portal messages using large language models and knowledge graph-based retrieval-augmented generation.	*J. Am. Med. Inform. Assoc.* **32**, 1032–1039 (2025). https://doi.org/10.1093/jamia/ocaf059	Ineligible study design
16	Megerian, J. T. et al.	Evaluation of an artificial intelligence-based medical device for diagnosis of autism spectrum disorder.	*npj Digit. Med.* **5**, 57 (2022). https://doi.org/10.1038/s41746-022-00598-6	Ineligible intervention
17	Morgan, J. I. & Muskett, T.	Interactional misalignment in the UK NHS 111 healthcare telephone triage service.	*Int. J. Med. Inform.* **134**, 104030 (2020). https://doi.org/10.1016/j.ijmedinf.2019.104030	Ineligible outcomes
18	Nabaweesi, R., Muthuka, J. K., Leo, J. & Levin, E.	Artificial intelligence in risk assessment and prevention.	*Prim. Care Clin. Off. Pract.* **52**, 687–697 (2025). https://doi.org/10.1016/j.pop.2025.07.002	Ineligible publication type
19	Ortega Páez, E. et al.	Is it possible to offer a clinical decision algorithm in COVID-19 disease in primary care?	*Pediatr. Aten. Primaria* **22**, 423–433 (2020).	Ineligible intervention
20	Park, H., Shin, D.-S., Wade, C. & Duong, D. B.-L.	Scoping review of nurse triage in primary care.	*BMC Nurs.* **24**, 1104 (2025). https://doi.org/10.1186/s12912-025-03740-3	Ineligible publication type
21	Payne, R. et al.	Patient safety in remote primary care encounters: multimethod qualitative study combining Safety I and Safety II analysis.	*BMJ Qual. Saf.* **33**, 573–586 (2024). https://doi.org/10.1136/bmjqs-2023-016674	Ineligible intervention
22	Rono, H. et al.	Effectiveness of an mHealth system on access to eye health services in Kenya: a cluster-randomised controlled trial.	*Lancet Digit. Health* **3**, e414–e424 (2021). https://doi.org/10.1016/S2589-7500(21)00083-2	Ineligible intervention

#	First author(s)	Title	Source	Reason for exclusion
23	Siira, E., Tyskbo, D. & Nygren, J.	Healthcare leaders' experiences of implementing artificial intelligence for medical history-taking and triage in Swedish primary care: an interview study.	*BMC Prim. Care* **25**, 268 (2024). https://doi.org/10.1186/s12875-024-02516-z	Ineligible outcomes
24	Spasić, I. & Button, K.	Patient triage by topic modeling of referral letters: feasibility study.	*JMIR Med. Inform.* **8**, e21252 (2020). https://doi.org/10.2196/21252	Ineligible intervention
25	Steerling, E., Svedberg, P., Nilsen, P., Siira, E. & Nygren, J.	Influences on trust in the use of AI-based triage — an interview study with primary healthcare professionals and patients in Sweden.	*Front. Digit. Health* **7**, 1565080 (2025). https://doi.org/10.3389/fdgth.2025.1565080	Ineligible outcomes
26	Tuan, W.-J., Sehgal, N. & Zgierska, A. E.	Using electronic health record's data to assess daily dose of opioids prescribed for outpatients with chronic non-cancer pain.	*Fam. Med. Community Health* **9**, e001277 (2021). https://doi.org/10.1136/fmch-2021-001277	Ineligible intervention
27	Wang, B., Li, W., Bradlow, A., Bazuaye, E. & Chan, A. T. Y.	Improving triaging from primary care into secondary care using heterogeneous data-driven hybrid machine learning.	*Decis. Support Syst.* **166**, 113899 (2023). https://doi.org/10.1016/j.dss.2022.113899	Ineligible setting

Exclusion categories: *Ineligible intervention* (not AI-enabled triage; e.g., administrative tool, no triage function, or no AI component); *Ineligible outcomes* (no safety, accuracy, or equity outcomes reported); *Ineligible publication type* (review, protocol, editorial, commentary, or abstract only); *Ineligible study design* (retrospective record review, simulation, vignette, or laboratory study without real patient encounters); *Ineligible setting* (not primary care; e.g., hospital, emergency department, or specialist setting only).
