# Supplementary material for: Patient safety and health equity of AI-enabled triage tools in primary care: a systematic review
Source: BMJ Health Care Inform. 2026 Oct 28;33(1):e102427. doi: 10.1136/bmjhci-2026-102427 (PMC13723792; doi:10.1136/bmjhci-2026-102427)
Supplement: Supplementary file 7 [file bmjhci-33-1-s007.pdf]

Online supplemental file 7

QUADAS-2 Supplementary Sensitivity Analysis

Patient Safety and Health Equity of AI-Enabled Triage Tools in Primary Care: A Systematic Review
PROSPERO Registration: CRD420251179046

Online supplemental file 7

QUADAS-2 Sensitivity Analysis of Diagnostic Accuracy Studies

Patient Safety and Health Equity of AI-Enabled Triage Tools in Primary Care: A Systematic Review

PROSPERO CRD420251179046

Aymn Alamoudi1,2, Yanying Gai1, Salwa S. Zghebi1, Evangelos Kontopantelis3,4, Benjamin C Brown1

[1] Division of Population Health, Health Services Research and Primary Care, School of Health Sciences, Faculty of Biology, Medicine and Health, University of Manchester, Manchester, United Kingdom

[2] Department of Public Health, School of Nursing and Health Sciences, Jazan University, Jazan, Saudi Arabia

[3] Division of Informatics, Imaging and Data Sciences, School of Health Sciences, Faculty of Biology, Medicine and Health, University of Manchester, Manchester, United Kingdom

[4] Division of Family Medicine, Yong Loo Lin School of Medicine, National University of Singapore, Singapore

ORCID: A.A. 0000-0001-8101-8218; Y.G. 0009-0000-2224-4952; S.S.Z. 0000-0002-7978-1094; E.K. 0000-0001-6450-5815; B.C.B. 0000-0001-9975-4782

1. Application of QUADAS-2

The Quality Assessment of Diagnostic Accuracy Studies tool, version 2 (QUADAS-2)[1] was applied as a supplementary sensitivity analysis to the three included studies that evaluate AI-triage accuracy against a clinician-judgement reference standard (Liu 2024[4], Chan 2021[5], Verzantvoort 2018[6]). Judson 2020[7] was not assessed with QUADAS-2 because its outcome is care-utilisation behaviour at 48 hours rather than a clinician-judgement reference standard. The Mixed Methods Appraisal Tool (MMAT) version 2018[2] remains the pre-registered primary quality-appraisal tool for the review (PROSPERO CRD420251179046); QUADAS-2 provides additional domain-specific scrutiny of patient selection, index test, reference standard, and flow and timing that is not captured by MMAT's broader criteria.

The primary QUADAS-2 assessment was performed by the first reviewer (AA) against the full text of each study and independently verified by the second reviewer (YG). The pre-specified procedure was to resolve any discrepancies by discussion, with adjudication by a third reviewer (SSZ) where required. Judgments are reported using standard QUADAS-2 terminology (Low / High / Unclear).

2. QUADAS-2 Tool as Applied

The standard QUADAS-2 tool[1] was applied without modification. The signalling questions answered for each domain in each study are listed in Table SM7.1 for reference. Domain-level Risk of Bias was rated Low when all signalling questions were answered "Yes", High when any answer was "No", and Unclear when any answer was "Unclear" and none was "No". Detailed judgments with supporting evidence are provided in Section 4.

Table SM7.1 — QUADAS-2 signalling questions applied across all included studies.

Domain	Signalling questions (answered Yes / No / Unclear)
**Domain 1 Patient**	1.1 Was a consecutive or random sample of patients enrolled?

**Selection**	1.2 Was a case-control design avoided?<br>1.3 Did the study avoid inappropriate exclusions?
**Domain 2 Index Test**	2.1 Were the index test results interpreted without knowledge of the results of the reference standard?<br>2.2 If a threshold was used, was it pre-specified?
**Domain 3 Reference Standard**	3.1 Is the reference standard likely to correctly classify the target condition?<br>3.2 Were the reference standard results interpreted without knowledge of the results of the index test?
**Domain 4 Flow and Timing**	4.1 Was there an appropriate interval between index test and reference standard?<br>4.2 Did all patients receive a reference standard?<br>4.3 Did all patients receive the same reference standard?<br>4.4 Were all patients included in the analysis?

3. Summary Traffic Light Table

Detailed judgments with supporting evidence are provided in Section 4. Applicability concerns with detailed rationales are provided in Section 5.

Table SM7.2 — Summary traffic-light overview of QUADAS-2 Risk of Bias judgments.

Colour coding: green = low risk of bias; yellow = unclear; red = high risk of bias.

Study	D1 Patient Selection	D2 Index Test	D3 Reference Standard	D4 Flow and Timing	Overall
**Liu 2024**	**High**	**Low**	**Unclear**	**Low**	**High**
**Chan 2021**	**High**	**Low**	**High**	**Unclear**	**High**
**Verzantvoort 2018**	**High**	**Low**	**Unclear**	**High**	**High**

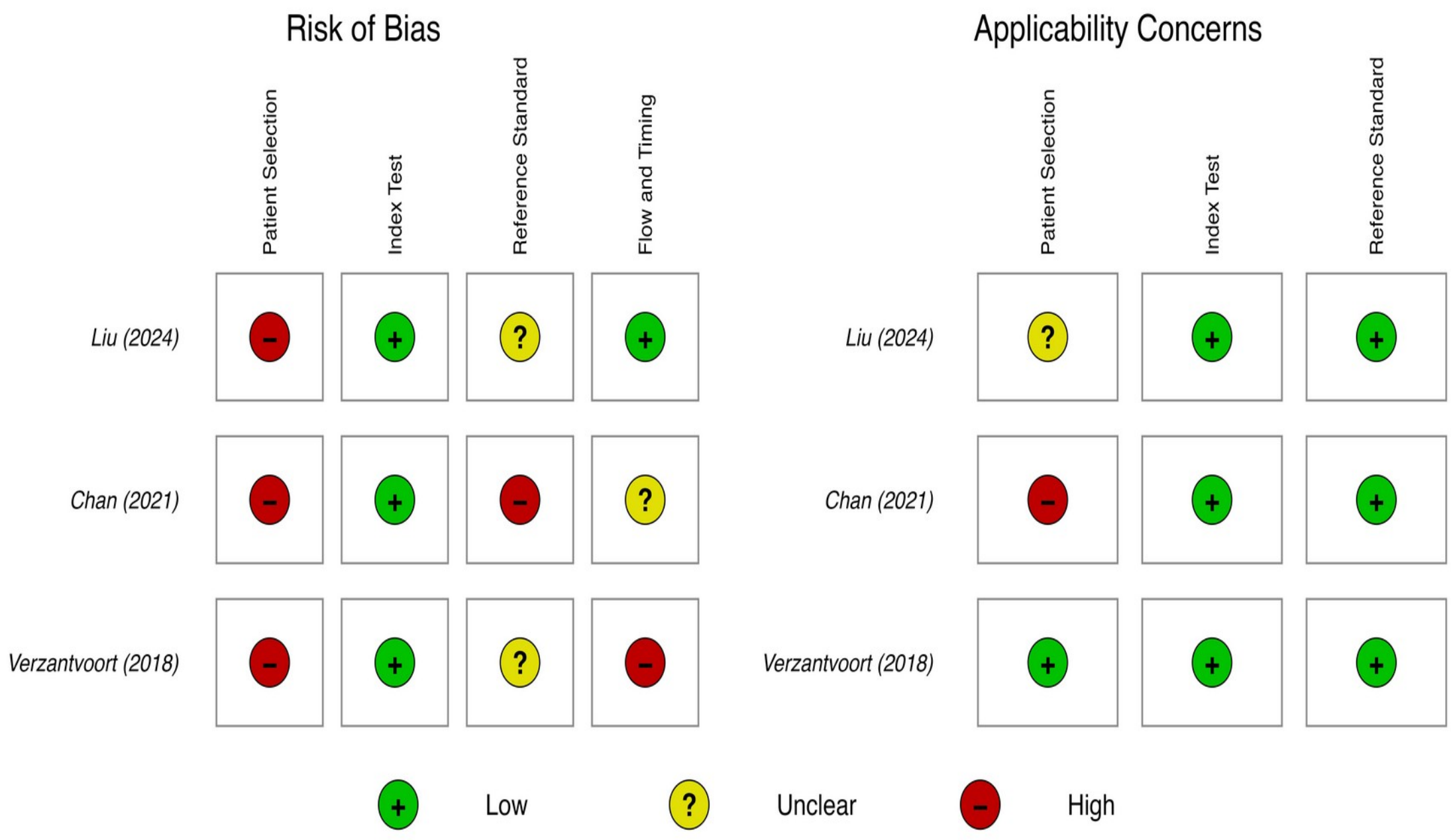

***Figure SM7.1 — Summary traffic-light figure of QUADAS-2 Risk of Bias and Applicability Concerns for the three diagnostic-accuracy studies, generated using the robvis Shiny application*[3].**

4. Domain-Level Risk of Bias Judgments with Supporting Evidence

One representative verbatim quotation is provided per domain, drawn from the published peer-reviewed article.

Table SM7.3 — Domain-level Risk of Bias judgments with verbatim supporting evidence.

Study	Domain	RoB	Key Evidence (brief rationale + one verbatim quote from source)
**Liu 2024**	D1 Patient Selection	**High**	Three explicit exclusions acknowledged by authors in Discussion: age <18 (tool designed for ≥15), digital competence, and waiting-room severity skew. **Quote:** *“only individuals over the legal age of 18 years were recruited”*
	D2 Index Test	**Low**	Automated ESC with pre-specified MSAH/THL thresholds; nurses blinded to ESC output until completing their own assessment. **Quote:** *“The triage nurses did not get to know the result of the ESC”*
	D3 Reference Standard	**Unclear**	119 different nurses with no inter-rater reliability check; authors acknowledge sick-leave-related reference standard bias. **Quote:** *“potentially biasing the calculated ESC accuracy”*
	D4 Flow and Timing	**Low**	Same-visit interval; all 877 collected cases

Study	Domain	RoB	Key Evidence (brief rationale + one verbatim quote from source)
			included in analysis; uniform reference standard applied. **Quote:** *"877 patient user assessment cases were successfully collected"*
**Chan 2021**	D1 Patient Selection	**High**	Self-selection via waiting-room kiosk with financial incentive; authors acknowledge systematic under-recruitment of severely unwell patients (ambulance bypass). **Quote:** *"incentivized to participate with opportunities to win $50 gift cards"*
	D2 Index Test	**Low**	Automated rule-based decision tree; patients blinded to output and treating physicians blinded to participation. **Quote:** *"treating physicians were blinded to patient participation"*
	D3 Reference Standard	**High**	Algorithm designer (author FC) also categorised the reference standard; categorisation schema in Table 1 maps "Urgency" to "Hospital or primary care" (two acceptable outputs for urgency cases). **Quote:** *"physician authors FC and SL and, by consensus, assigned an appropriate categorization"*
	D4 Flow and Timing	**Unclear**	135 mismatch exclusions (~19% of analysed sample); justification methodologically defensible but post-hoc nature warrants Unclear. **Quote:** *"symptom checker responses were significantly mismatched for 135 patients"*
**Verzantvoort 2018**	D1 Patient Selection	**High**	Accuracy subsample 126/4,456 (2.8%) via 4-stage self-selection; Group 2 contained significantly more males (48% vs 34%; p=0.02). **Quote:** *"A convenience sample of users was phoned by a triage nurse"*
	D2 Index Test	**Low**	Automated smartphone app with pre-specified NTS-based thresholds; CE-labelled Class I medical device. **Quote:** *"registered as a Class I medical device"*
	D3 Reference Standard	**Unclear**	Reference standard (nurse telephone triage using NTS) is appropriate in principle (Q3.1 = Yes), but the study does not report whether triage nurses were blinded to the app's specific recommendation during their assessment (Q3.2 = Unclear). **Quote: "Telephone triage was regarded as the reference standard"**
	D4 Flow and Timing	**High**	9% of phoned users contacted the day after app use; 17/143 reachable excluded (12%); authors attribute mismatches to timing delay. **Quote:** *"This delay has probably resulted in mismatches"*

5. Applicability Concerns

Applicability assesses whether the patient population, index test, and reference standard in each study match the review question. In QUADAS-2, Applicability and Risk of Bias are independent dimensions: a study may carry High Risk of Bias (because of how its methods were applied) while remaining Low for Applicability (because its population, intervention, and comparator are conceptually appropriate to the review question), and vice versa. Among the three included studies, Verzantvoort 2018's at-home smartphone use most closely matches the patient-initiated remote-use scenario the review targets; Liu 2024's waiting-room completion occupies an intermediate position; and Chan 2021's in-clinic kiosk completion (with patients who had already decided to seek in-person care) departs furthest from this scenario.

Table SM7.4 — Applicability concerns with domain-level rationale across the three QUADAS-2 applicability domains.

Study	Domain	Rating	Rationale
**Liu 2024**	D1 Patient Selection	**Unclear**	The waiting-room completion context occupies an intermediate position between Verzantvoort's at-home smartphone use (closely matching the patient-initiated remote-use scenario the review targets) and Chan's in-clinic kiosk completion (which patients reached only after deciding to seek in-person care). Whether triage decision-making in a waiting-room differs systematically from at-home pre-contact triage cannot be determined from the available data; the Unclear rating reflects this uncertainty rather than evidence of mismatch.
	D2 Index Test	**Low**	The Omaolo electronic symptom checker is a CE-marked Class IIa medical device deployed nationally in Finnish primary care; it represents the type of AI-enabled triage tool targeted by the review.
	D3 Reference Standard	**Low**	Nurse triage assessment is the standard-of-care reference for evaluating symptom-checker triage accuracy in primary care and is appropriate for the review question.
**Chan 2021**	D1 Patient Selection	**High**	The evaluation context — kiosk-based completion in emergency department and family physician waiting rooms by patients who had already decided to seek in-person care — does not match the patient-initiated remote-use scenario the review targets. The tool's triage advice was generated retrospectively to an in-person care decision the patient had already made, rather than informing whether and where to seek care.
	D2 Index Test	**Low**	The prototype rule-based decision tree

Study	Domain	Rating	Rationale
			represents the algorithmic approach typical of AI-enabled triage tools assessed in the review.
	D3 Reference Standard	**Low**	Treating physicians' diagnoses and management plans constitute a conceptually appropriate reference standard for triage accuracy in primary care and align with the review question. The High Risk of Bias rating in Section 4 reflects how the reference standard was applied (algorithm-designer involvement in categorisation, categorisation schema permitting multiple acceptable outputs); it does not indicate that the reference standard itself is unsuitable for the review question. Risk of Bias and Applicability address independent dimensions in QUADAS-2.
**Verzantvoort 2018**	D1 Patient Selection	**Low**	The smartphone application was used by real out-of-hours primary care help-seekers at home before any contact with healthcare professionals, matching the patient-initiated remote-use scenario the review targets more closely than either Liu's waiting-room context or Chan's in-clinic kiosk. The High Risk of Bias rating in Section 4 reflects the selection mechanism of the 126-patient accuracy subsample (four sequential self-selection filters reducing the sample to 2.8% of survey respondents); this is a methodological concern about how the accuracy subsample was constituted, not a concern about whether the population matches the review question. The two ratings address independent dimensions in QUADAS-2.
	D2 Index Test	**Low**	The "Should I see a doctor?" smartphone app is a CE-labelled medical device for patient-initiated triage, matching the review definition.
	D3 Reference Standard	**Low**	Nurse telephone triage using the Dutch Triage System is a conceptually appropriate reference standard for primary care triage accuracy in the Dutch OOH context. The Unclear Risk of Bias rating in Section 4 reflects unreported blinding of nurses to the app's specific recommendation; this concerns how the reference standard was applied in this study, not whether nurse telephone triage is in principle a suitable reference standard for the review question.

6. Comparison with MMAT

The two tools were substantially aligned in identifying methodological concerns, but diverged on specific diagnostic-accuracy issues. Concerns flagged by QUADAS-2 that were not captured by MMAT include reference-standard conflict of interest in Chan 2021, categorisation-schema structural advantage in Chan 2021, and unreported blinding of the reference-standard assessor in Verzantvoort 2018. These are not deficiencies of MMAT but illustrate the value of a diagnostic-accuracy-specific tool for this review's subset of accuracy studies.

Table SM7.5 — Concordance of MMAT (primary) and QUADAS-2 (supplementary) ratings, with key methodological divergences.

Study	MMAT (primary)	QUADAS-2 Overall RoB	Key divergence
**Liu 2024**	4/5 — Can't tell on 3.4	**High**	QUADAS-2 identified three explicit patient-selection exclusions documented in Discussion limitations (not captured by MMAT's measurement focus).
**Chan 2021**	5/5 (High) — all Yes	**High**	QUADAS-2 identified reference-standard conflict (algorithm designer FC also categorised reference standard) and categorisation schema permitting two output levels for urgency cases.
**Verzantvoort 2018**	4/5 — Can't tell on 3.3	**High**	QUADAS-2 identified that nurse blinding to the app's specific recommendation was not explicitly reported (Q3.2 = Unclear), an issue not captured by MMAT's broader measurement criteria.

Abbreviations: AA = Aymn Alamoudi; MSAH = Finnish Ministry of Social Affairs and Health; QUADAS-2 = Quality Assessment of Diagnostic Accuracy Studies 2; RoB = risk of bias; SSZ = Salwa S. Zghebi; THL = Finnish Institute for Health and Welfare; YG = Yanying Gai.

7. References

1. Whiting, P. F. et al. QUADAS-2: a revised tool for the quality assessment of diagnostic accuracy studies. Ann. Intern. Med. 155, 529–536 (2011).
2. Hong, Q. N. et al. The Mixed Methods Appraisal Tool (MMAT) version 2018 for information professionals and researchers. Educ. Inf. 34, 285–291 (2018).
3. McGuinness, L. A. & Higgins, J. P. T. Risk-of-bias VISualization (robvis): An R package and Shiny web app for visualising risk-of-bias assessments. Res. Synth. Methods 12, 55–61 (2021).
4. Liu, V., Kaila, M. & Koskela, T. Triage accuracy and the safety of user-initiated symptom assessment with an electronic symptom checker in a real-life setting: instrument validation study. JMIR Hum. Factors 11, e55099 (2024).
5. Chan, F. et al. Performance of a new symptom checker in patient triage: Canadian cohort study. PLoS One 16, e0260696 (2021).
6. Verzantvoort, N. C. M., Teunis, T., Verheij, T. J. M. & van der Velden, A. W. Self-triage for acute primary care via a smartphone application: practical, safe and efficient? PLoS One 13, e0199284 (2018).

7. Judson, T. J. et al. Rapid design and implementation of an integrated patient self-triage and self-scheduling tool for COVID-19. J. Am. Med. Inform. Assoc. 27, 860–866 (2020).
