# Supplementary material for: Patient safety and health equity of AI-enabled triage tools in primary care: a systematic review
Source: BMJ Health Care Inform. 2026 Oct 28;33(1):e102427. doi: 10.1136/bmjhci-2026-102427 (PMC13723792; doi:10.1136/bmjhci-2026-102427)
Supplement: Supplementary file 8 [file bmjhci-33-1-s008.pdf]

Supplementary Material 8

Meta-Analysis Raw Data and R Code

Patient Safety and Health Equity of AI-Enabled Triage Tools in Primary Care: A Systematic Review

PROSPERO Registration: CRD420251179046

Supplementary Material 8

Meta-analysis: raw data, statistical methods, and reproducible R code

Patient Safety and Health Equity of AI-Enabled Triage Tools in Primary Care: A Systematic Review
PROSPERO: CRD420251179046

Aymn Alamoudi[1,2], **Yanying Gai**[1], **Salwa S. Zghebi**[1], **Evangelos Kontopantelis**[3,4], **Benjamin Brown**[1]

[1] *Division of Population Health, Health Services Research and Primary Care, School of Health Sciences, Faculty of Biology, Medicine and Health, University of Manchester, Manchester, United Kingdom*

[2] *Department of Public Health, School of Nursing and Health Sciences, Jazan University, Jazan, Saudi Arabia*

[3] *Division of Informatics, Imaging and Data Sciences, School of Health Sciences, Faculty of Biology, Medicine and Health, University of Manchester, Manchester, United Kingdom*

[4] *Division of Family Medicine, Yong Loo Lin School of Medicine, National University of Singapore, Singapore*

ORCID: A.A. 0000-0001-8101-8218; Y.G. 0009-0000-2224-4952; S.S.Z. 0000-0002-7978-1094; E.K. 0000-0001-6450-5815; B.B. 0000-0001-9975-4782

---

1. Overview

This Supplementary Material provides the raw data, statistical methods, and reproducible R code used for the random-effects meta-analysis reported in the main manuscript. The meta-analysis pooled three studies with comparable proportion-based outcomes against clinical reference standards: Liu 2024, Chan 2021 (primary care subgroup), and Verzantvoort 2018. Judson 2020 was excluded from the meta-analysis because it used subsequent 48-hour care utilisation rather than a clinical comparator as its reference standard, which differs from the comparators used by the other three studies.

2. Protocol deviations

During manuscript revision, two deviations from the registered protocol were introduced and are reported transparently here. First, the protocol pre-specified narrative synthesis only; a random-effects meta-analysis was added for three studies that reported comparable proportion-based outcomes against clinical reference standards. The decision was taken in light of the retrieved studies and the methodological feasibility of pooling, and is reported in this Supplementary Material together with the full R code, raw data, and pooled results.

Second, the protocol specified GRADE-CERQual for certainty assessment. GRADE-CERQual is designed for qualitative evidence syntheses, and the included primary studies were quantitative diagnostic-accuracy studies. Standard GRADE was also not applied because the small number of clinically and methodologically heterogeneous studies and the largely narrative synthesis approach precluded meaningful application. Risk of bias and methodological quality are reported using the Mixed Methods Appraisal Tool (primary) and QUADAS-2 (sensitivity analysis) in Supplementary Materials 4 and 7 respectively.

3. Raw event counts and denominators

Table SM8.1 presents the event counts (numerators) and total sample sizes (denominators) extracted from the three included studies for each meta-analysed outcome. All values were verified against the original study publications during data extraction (see main manuscript Methods, Data extraction).

Study	Outcome	Events	Total	Proportion
Liu 2024	Safe triage	856	877	0.9761
Liu 2024	Under-triage	151	877	0.1722
Liu 2024	Over-triage	255	877	0.2908
Chan 2021 (PC)	Safe triage	255	300	0.8500
Chan 2021 (PC)	Under-triage	1	300	0.0033
Chan 2021 (PC)	Over-triage	20	300	0.0667
Verzantvoort 2018	Safe triage	102	126	0.8095
Verzantvoort 2018	Under-triage	14	126	0.1111
Verzantvoort 2018	Over-triage	10	126	0.0794

Table SM8.1. *Raw event counts and denominators for each included study and meta-analysed outcome. PC = primary care subgroup. Total combined sample: n = 1,303 patients.*

4. Statistical methods

4.1 Effect measure

The effect measure was a single proportion for each outcome (safe triage, under-triage, over-triage). Proportions were transformed using the logit transformation prior to pooling, which is appropriate for proportions that are not extreme and avoids known boundary issues with the Freeman-Tukey double arcsine transformation when proportions approach 0 or 1.

4.2 Pooling method

A random-effects model was fitted using the inverse variance method on the logit scale. Between-study variance (τ^2) was estimated using the restricted maximum-likelihood (REML) estimator. The Hartung-Knapp-Sidik-Jonkman[3–5] (HKSJ) adjustment was applied to confidence intervals to account for uncertainty in τ^2 estimation, which is particularly important given the small number of included studies (k = 3).

4.3 Heterogeneity

Statistical heterogeneity was quantified using I^2 and τ^2[6]. Prediction intervals were not reported because, with only three studies per outcome (k = 3), τ^2 is estimated on one degree of freedom, and the resulting interval bounds are uninformative.

4.4 Funnel plot asymmetry and publication bias

Publication bias was not formally assessed: in the absence of an intervention contrast, asymmetry tests have limited interpretive value, and the small number of pooled studies (k = 3) further limits statistical power.

4.5 Software

All analyses were conducted in R (version 4.4.2)[1] using the *meta* package (version 8.2-1)[2]. Forest plots were generated using the forest() function in the *meta* package and combined into a multi-panel supplementary figure (presented at the end of this file) using the *magick* R package.

5. Reproducible R code

The complete R script below reproduces the meta-analysis. Running this code in R (≥ 4.0) with the meta and magick packages installed will generate all pooled estimates, confidence intervals, heterogeneity statistics, and forest plots presented at the end of this file.

```
# ============================================================
# AI Triage Safety - Meta-analysis of Proportions
# Studies: Liu 2024, Chan 2021 (PC subgroup), Verzantvoort 2018
# Model: Random-effects (REML) with Hartung-Knapp-Sidik-Jonkman
# Software: R 4.4.2, meta package 8.2-1, magick R package
# ============================================================

# Install packages (first time only)
# install.packages(c("meta", "magick"))
library(meta)
library(magick)

# --- Raw data ----------------------------------------------
studies <- data.frame(
  study        = c("Liu 2024", "Chan 2021 (PC)", "Verzantvoort 2018"),
  safe_events  = c(856, 255, 102),
  safe_total   = c(877, 300, 126),
  under_events = c(151,   1,  14),
  under_total  = c(877, 300, 126),
  over_events  = c(255,  20,  10),
  over_total   = c(877, 300, 126)
)

# --- Meta-analysis objects ----------------------------------
ma_safe <- metaprop(
  event            = safe_events,
  n                = safe_total,
  studlab          = study,
  data             = studies,
  method           = "Inverse",
  sm               = "PLOGIT",
  method.tau       = "REML",
  method.random.ci = "HK"
)

ma_under <- metaprop(
  event            = under_events,
  n                = under_total,
  studlab          = study,
  data             = studies,
  method           = "Inverse",
  sm               = "PLOGIT",
  method.tau       = "REML",
  method.random.ci = "HK"
)
```

```r
ma_over <- metaprop(
  event            = over_events,
  n                = over_total,
  studlab          = study,
  data             = studies,
  method           = "Inverse",
  sm               = "PLOGIT",
  method.tau       = "REML",
  method.random.ci = "HK"
)

# --- Forest plot helper ---------------------------------------
# Renders one panel with random-effects diamond only (common-effect
# diamond, prediction interval, and heterogeneity stats are hidden),
# then adds a bold caption beneath the plot.
generate_forest <- function(meta_obj, filename, smlab_text, bottom_label,
                            xlim_vals = c(0, 1)) {
  png(filename, width = 4600, height = 1500, res = 300)
  forest(meta_obj,
         xlim       = xlim_vals,
         plotwidth  = "7cm",
         digits     = 3,
         prediction = FALSE,
         common     = FALSE,
         random     = TRUE,
         col.square  = "black",
         col.diamond = "black",
         fontfamily  = "sans",
         leftcols   = c("studlab", "event", "n"),
         leftlabs   = c("Study", "Events", "Total"),
         rightcols  = c("effect", "ci", "w.random"),
         rightlabs  = c("Proportion", "95% CI", "Weight"),
         smlab      = smlab_text,
         hetstat    = FALSE,
         addrow     = TRUE)
  dev.off()
  img  <- image_read(filename)
  info <- image_info(img)
  img <- image_extent(
    img,
    geometry = paste0(info$width, "x", info$height + 120),
    gravity  = "north",
    color    = "white"
  )
  img <- image_annotate(
    img,
    text     = bottom_label,
    gravity  = "south",
    location = "+0+50",
    size     = 80,
    weight   = 700,
    color    = "black"
  )
  image_write(img, filename)
}
```

```r
# --- Generate the three panels -------------------------------
generate_forest(ma_safe,  "forest_safe.png",
                smlab_text   = "Safe triage proportion",
                bottom_label = "(a) Safe triage",
                xlim_vals    = c(0, 1))
generate_forest(ma_under, "forest_under.png",
                smlab_text   = "Under-triage proportion",
                bottom_label = "(b) Under-triage",
                xlim_vals    = c(0, 0.30))
generate_forest(ma_over,  "forest_over.png",
                smlab_text   = "Over-triage proportion",
                bottom_label = "(c) Over-triage",
                xlim_vals    = c(0, 0.40))

# --- Combine the three panels into a multi-panel supplementary figure ---
img_a <- image_read("forest_safe.png")
img_b <- image_read("forest_under.png")
img_c <- image_read("forest_over.png")
combined <- image_append(c(img_a, img_b, img_c), stack = TRUE)
image_write(combined, "Figure_2_MetaAnalysis_Combined.png",  format = "png")
image_write(combined, "Figure_2_MetaAnalysis_Combined.tiff", format = "tiff")

# Sensitivity analyses: harmonised threshold + leave-one-out
library(meta)
studies <- c("Liu 2024", "Chan 2021 (PC)", "Verzantvoort 2018")
n       <- c(877, 300, 126)
under   <- c(61,   7,  14)
over    <- c(107, 23,  10)
concord <- n - under - over
run_pooled <- function(events, label) {
  m <- metaprop(event = events, n = n, studlab = studies,
                sm = "PLOGIT", method = "Inverse",
                method.tau = "REML", hakn = TRUE)
  cat("\n=====", label, "=====\n"); print(summary(m))
  cat("\n--- Leave-one-out:", label, "---\n")
  print(metainf(m, pooled = "random"))
  invisible(m)
}
m_concord <- run_pooled(concord, "Binary disposition concordance")
m_under   <- run_pooled(under,   "Directional under-triage")
m_over    <- run_pooled(over,    "Directional over-triage")
png("SM8_Sensitivity_Harmonised_Forest.png", width = 2400, height = 3200, res = 300)
par(mfrow = c(3, 1))
forest(m_concord, main = "Binary disposition concordance (harmonised threshold)")
forest(m_under,   main = "Directional under-triage (harmonised threshold)")
forest(m_over,    main = "Directional over-triage (harmonised threshold)")
dev.off()
sessionInfo()
```

6. Pooled results

Table SM8.2 summarises the pooled estimates from the random-effects model with Hartung-Knapp-Sidik-Jonkman adjustment, alongside heterogeneity statistics.

Outcome	Random-effects (95% CI)	I^2	τ^2
**Safe triage**	0.908 (0.319 – 0.995)	97.0%	1.46
**Under-triage**	0.050 (0.000 – 0.920)	89.7%	4.05
**Over-triage**	0.123 (0.012 – 0.610)	97.1%	0.92

Table SM8.2. *Random-effects pooled proportions with heterogeneity statistics. Confidence intervals incorporate the Hartung-Knapp-Sidik-Jonkman adjustment.*

7. Notes on interpretation

Pooled estimates should be interpreted as weighted summaries across studies with differing operational definitions, reference standards, and clinical settings, rather than as precise estimates of a single underlying parameter. Specifically:

- Safe triage was retained as defined by each primary study. Liu operationalised safe triage as exactly matched, overly conservative, or safe-but-underconservative compared to nurse triage; Chan operationalised it as accurate triage recommendation against physician diagnosis in the primary care subgroup; Verzantvoort operationalised it as app advice corresponding to telephone nurse triage.
- Under-triage definitions differed substantially: Chan limited the definition to missed urgencies causing potential harm (resulting in a very low rate of 0.3%), while Liu counted any downward discrepancy from nurse triage (17.2%).
- Over-triage definitions also varied, contributing to the observed heterogeneity.

These interpretive caveats are discussed in the main manuscript (Limitations section).

Abbreviations: CI = confidence interval; HKSJ = Hartung-Knapp-Sidik-Jonkman; REML = restricted maximum likelihood.

8. Sensitivity analyses: harmonised threshold and leave-one-out

The three studies' graded urgency categories cannot be aligned without individual participant data. Under- and over-triage were therefore re-derived at the one threshold supported by all three published datasets - professional clinical contact required versus self-care - harmonising both numerators and denominators, and re-pooled under the same model specification as the primary analysis (PLOGIT, inverse variance, REML, Hartung-Knapp), with leave-one-out as an influence check.

Study	Published categories	Allocated to 'professional contact'	Allocated to 'self-care'	Under-triage	Over-triage	Concordant	N
Liu 2024	P0–P4 and L2–L4	P0–P4 (and nurse-contact dispositions)	L2–L4	61	107	709	877
Chan 2021 (primary-care subgroup)	Emergency, urgent, routine, home care	Emergency, urgent, routine	Home care	7	23	270	300
Verzantvoort 2018	Call a doctor (GP or out-of-hours) vs don't call	Call a doctor	Don't call (self-care, wait-and-see)	14	10	102	126

Table SM8.3. *Category allocation and harmonised counts by study.*

Harmonised counts intentionally differ from each study's own definitions: Chan et al.'s harm-limited under-triage is 1/300, whereas the threshold-based re-derivation yields 7/300; Liu et al.'s 21 potentially unsafe assessments reflect their safety threshold, whereas the contact threshold yields 61/877.

Outcome	Pooled proportion (95% CI)	I^2	Leave-one-out range of point estimates
**Binary disposition concordance**	0.844 (0.649–0.940)	84.8%	0.809–0.862
**Directional under-triage**	0.059 (0.008–0.326)	83.6%	0.043–0.083
**Directional over-triage**	0.096 (0.047–0.186)	65.8%	0.078–0.108

Table SM8.4. *Harmonised pooled estimates (random effects, Hartung-Knapp) and leave-one-out ranges.*

Compared with the primary analysis, heterogeneity decreased and confidence intervals narrowed, with the direction of conclusions unchanged in every leave-one-out iteration. The harmonised estimates remain exploratory: re-deriving on a common definition removes the definitional layer of heterogeneity, while differences in settings and reference standards remain.

9. References

1. R Core Team. R: A language and environment for statistical computing. (R Foundation for Statistical Computing, 2024); https://www.R-project.org/.

2. Balduzzi, S., Rücker, G. & Schwarzer, G. How to perform a meta-analysis with R: a practical tutorial. Evid Based Ment Health 22, 153–160 (2019).

3. Hartung, J. & Knapp, G. On tests of the overall treatment effect in meta-analysis with normally distributed responses. Stat. Med. 20, 1771–1782 (2001).

4. Sidik, K. & Jonkman, J. N. A simple confidence interval for meta-analysis. Stat. Med. 21, 3153–3159 (2002).

5. IntHout, J., Ioannidis, J. P. A. & Borm, G. F. The Hartung-Knapp-Sidik-Jonkman method for random effects meta-analysis is straightforward and considerably outperforms the standard DerSimonian-Laird method. BMC Med. Res. Methodol. 14, 25 (2014).

6. Higgins, J. P. T. & Thompson, S. G. Quantifying heterogeneity in a meta-analysis. Stat. Med. 21, 1539–1558 (2002).

Forest plots

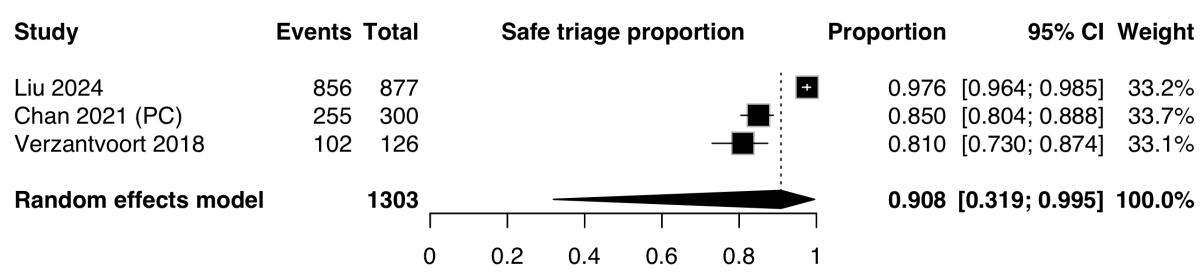

(a) Safe triage

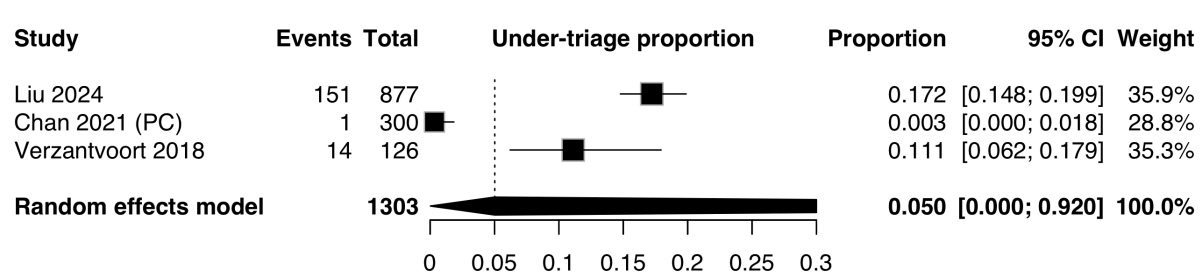

(b) Under-triage

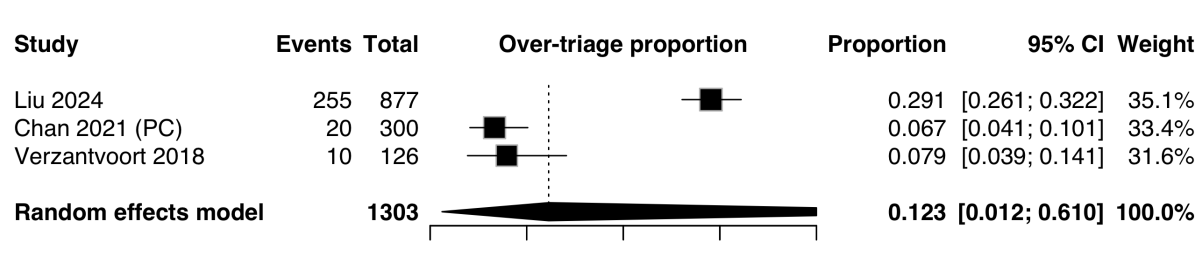

(c) Over-triage

Random-effects meta-analysis forest plots for three pooled outcomes across three included studies (Liu 2024, Chan 2021, Verzantvoort 2018; n = 1,303 patients). (a) Safe triage proportion: pooled estimate 0.908 (95% CI 0.319–0.995); I^2 = 97.0%; τ^2 = 1.46. (b) Under-triage proportion: pooled estimate 0.050 (95% CI 0.000–0.920); I^2 = 89.7%; τ^2 = 4.05. (c) Over-triage proportion: pooled estimate 0.123 (95% CI

0.012–0.610); I² = 97.1%; τ² = 0.92. Judson et al. was excluded from the meta-analysis because it used a different reference standard.

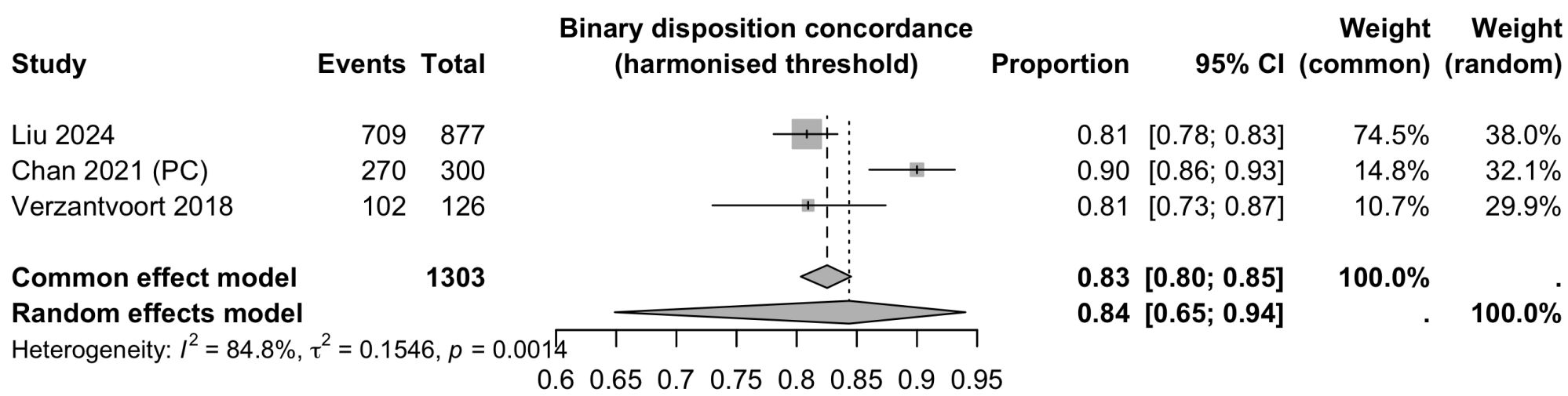

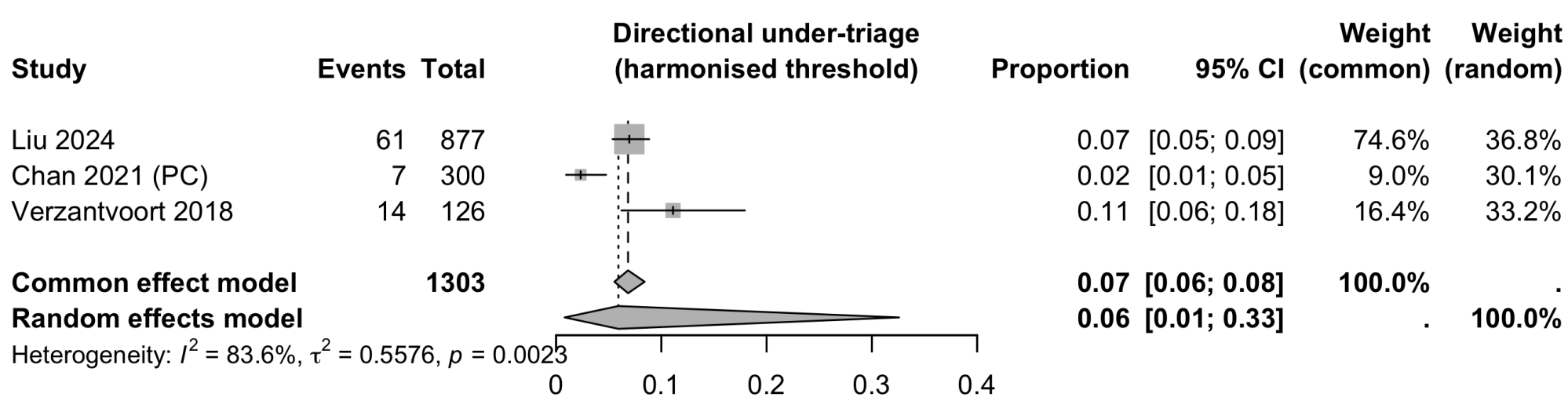

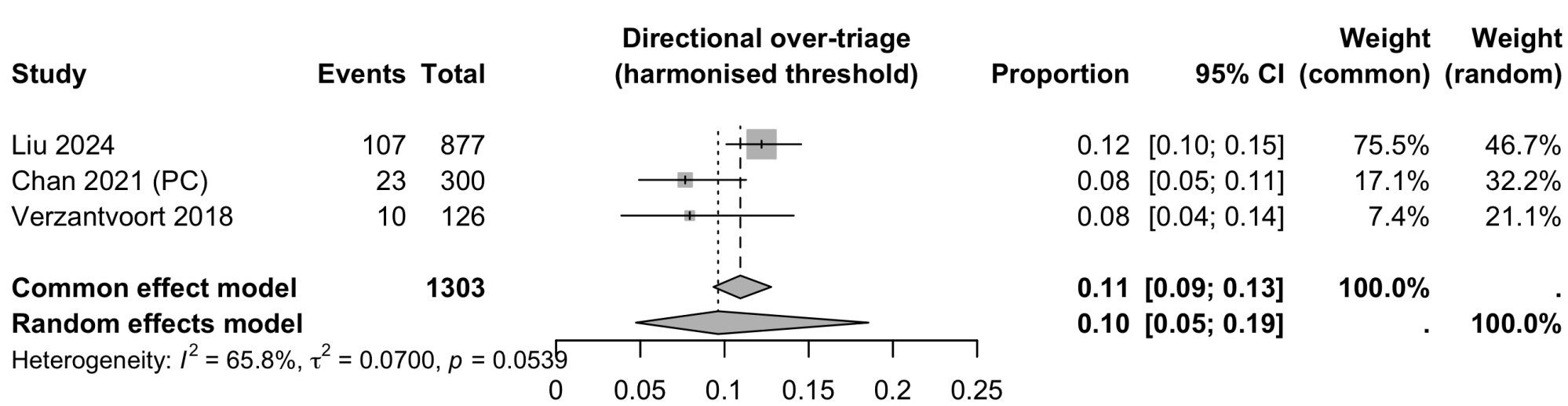

Sensitivity analysis forest plots (harmonised threshold): binary disposition concordance, directional under-triage, and directional over-triage.
